# Improved Metal-Free Approach for the Synthesis of Protected Thiol Containing Thymidine Nucleoside Phosphoramidite and Its Application for the Synthesis of Ligatable Oligonucleotide Conjugates

**DOI:** 10.3390/pharmaceutics15010248

**Published:** 2023-01-11

**Authors:** Zoltán Kupihár, Györgyi Ferenc, Vencel L. Petrovicz, Viktória R. Fáy, Lajos Kovács, Tamás A. Martinek, Zsófia Hegedüs

**Affiliations:** 1 Department of Medical Chemistry, University of Szeged, Dom ter 8., H-6720 Szeged, Hungary; 2 Institute of Plant Biology, Biological Research Centre, Eötvös Lóránd Research Network, H-6726 Szeged, Hungary; 3 ELKH-SZTE Biomimetic Systems Research Group, Eötvös Loránd Research Network, H-6720 Szeged, Hungary

**Keywords:** nucleoside synthesis, oligonucleotide conjugates, ligatable oligonucleotides, solid-phase conjugation, thiol-maleimide conjugation, templated ligation

## Abstract

Oligonucleotide conjugates are versatile scaffolds that can be applied in DNA-based screening platforms and ligand display or as therapeutics. Several different chemical approaches are available for functionalizing oligonucleotides, which are often carried out on the 5′ or 3′ end. Modifying oligonucleotides in the middle of the sequence opens the possibility to ligate the conjugates and create DNA strands bearing multiple different ligands. Our goal was to establish a complete workflow that can be applied for such purposes from monomer synthesis to templated ligation. To achieve this, a monomer is required with an orthogonal functional group that can be incorporated internally into the oligonucleotide sequence. This is followed by conjugation with different molecules and ligation with the help of a complementary template. Here, we show the synthesis and the application of a thiol-modified thymidine nucleoside phosphoramidite to prepare ligatable oligonucleotide conjugates. The conjugations were performed both in solution and on solid phase, resulting in conjugates that can be assembled into multivalent oligonucleotides decorated with tissue-targeting peptides using templated ligation.

## 1. Introduction

The ability of specifically recognizing complementary DNA strands makes oligonucleotides attractive tools for diagnostics, therapeutics, or platforms for displaying molecules. Decorating an oligonucleotide strand with molecules while keeping its ability to hybridize specifically to a target sequence has numerous applications. Oligonucleotides conjugated with cell-penetrating peptides, targeting moieties or antibodies, influence their pharmacokinetic properties that allow for their use as therapeutics or detection reagents [1,2,3,4]. Other applications exploit the specific hybridization to display molecules in a spatially defined arrangement, which then can be utilized as affinity reagents and protein surface mimetics [5,6,7], starting compounds for DNA-templated synthesis [8,9], or to create nanomaterials [10].

Many different synthetic approaches are available to functionalize oligonucleotide sequences [2,11,12,13], but these are mostly carried out on the 3′ or 5′ terminus. Modification within the sequence (termed internal modification) leaves both ends free, which opens the possibility to ligate the conjugates and create libraries of oligonucleotides with high number of modifications. Chemical approaches to prepare internally functionalized oligonucleotides through a modified nucleobase include conjugation using amide [6,7], thiol-maleimide [14,15], oxime bond [16] formation, Diels–Alder cycloaddition [17], and azide-alkyne click chemistry [18]. For the latter, phosphoramidites bearing alkyne groups are available to synthesize up to three different modifications in an oligonucleotide sequence [19]. Although these have great potential for further assembly, a limited number of applications have been shown in subsequent ligation reactions. The few examples include small molecule carboxylic acid [7,20] and peptide [21] functionalizations of amine-modified oligonucleotides that were subsequently ligated in the presence of a template and followed by in vitro evolution cycles to select protein binders [22,23].

Among all the available chemical approaches, an internal thiol functional group is the most versatile in terms of its use in several different synthetic pathways for conjugation [24,25,26] orthogonally to many functional groups. Internal thiol modification can be achieved using the commercially available S-Bz-thiol modifier C6 dT [27], but its susceptibility to degradation during ammonolysis disadvantageously requires deprotection in multiple steps, milder cleavage conditions, and does not allow selective deprotection. On the other hand, a thiol (RSH) protected as a *tert*-butyl disulfide (*t*-BuSSR) could overcome these issues [15,28,29,30,31,32,33]. The synthesis of cysteine-modified uridine with a *tert*-butyldisulfanyl (*t*-Bu-SS) protection group has been described and applied to conjugate peptides using native chemical ligation or thiol–maleimide chemistry, but its applicability in ligase catalysed templated ligation has not been investigated [14,34,35].

**Figure 1:**
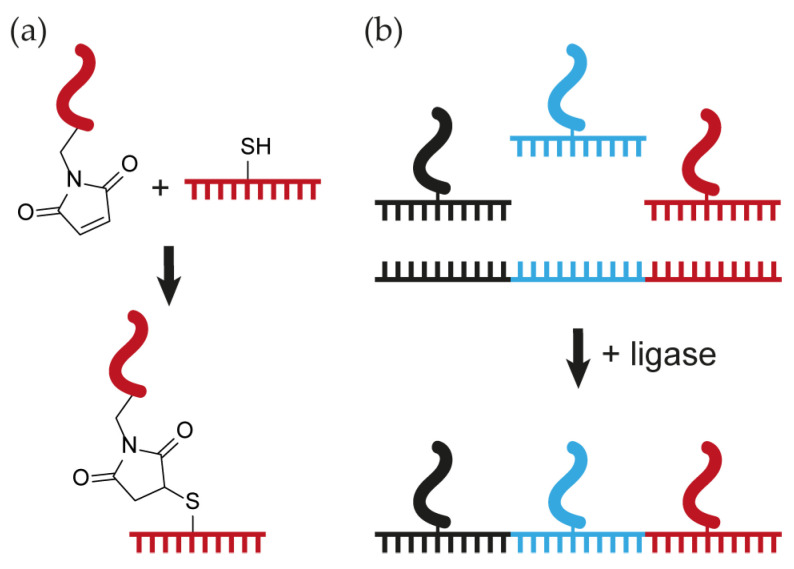
Scheme depicting our approach to prepare ligatable oligonucleotide conjugates. (**a**) Thiol–maleimide ligation using oligonucleotide with internal thiol modification conjugated to maleimide functionalized ligand and (**b**) ligase-catalyzed templated ligation of three different oligonucleotide conjugates, resulting in multifunctionalized DNA. S-shaped structure represents peptide sequence and comb-like structures belong to oligonucleotides.

Our goal was to establish a complete workflow to create DNA strands bearing multiple ligands through templated ligation using short ligatable oligonucleotide conjugates. To achieve this, the first step was the synthesis of a nucleoside that allows for the incorporation of an orthogonal functional group mid-sequence, leaving both oligonucleotide termini free. This is followed by the preparation of conjugates that can be ligated in the presence of a template. Here, we show the synthesis of *t*-Bu-SS-hymidine phosphoramidite (**11**) using a metal-free synthesis route. The monomer could be incorporated into oligonucleotide sequences and conjugated to maleimide-functionalized small molecules or peptides on solid and in solution phase. As the last step, we demonstrate the applicability of the conjugates in templated ligation to prepare a DNA strand bearing three different tissue-targeting peptide sequences (Figure 1).

## 2. Materials and Methods

### 2.1. General Remarks

The solvents and reagents for synthesis were obtained from Merck, TCI Europe, Alfa Aesar, or AK Scientific Inc. The 3′,5′-Bis-*O*-(*tert*-butyldimethylsilyl)thymidine (**2**) [36] and 3-[(pyridin-2-yl)disulfanyl]propanoic acid (**6**) [37] were prepared according to the indicated literature methods. Their characterization data were identical to the ones provided in the literature references. The systematic names of compounds were generated using the software ACD/Name 2021.2.0 (ACD/Labs).

The thin-layer chromatography used silica gel 60 F254 (Merck, Rahway, NJ, USA) and the column chromatography used silica gel 60, 40–63 μm (Merck). The ^1^H NMR spectra were recorded on a Bruker Ascend 500 MHz spectrometer (Bruker, Billerica, MA, USA) with a 5 mm BBO Prodigy Probe. The ^13^C NMR spectra and ^31^P NMR were recorded with the same instrument at 125 MHz and 200 MHz, respectively. The High-Resolution Mass Spectrometry analyzed data were measured on a Thermo Fisher Scientific Q Exactive Plus Orbitrap instrument equipped with a nano ESI ion source.

The reagents for oligonucleotide synthesis were obtained from Link Technologies, Sigma Aldrich, Hongene, and Molar Chemicals Kft. For peptide synthesis, Fmoc-protected amino acids were purchased from ChemImpex, GL Biochem, and Iris Biotech. The Tentagel R RAM was purchased from Rapp Polymere. The DMF used for peptide synthesis was purchased from VWR.

The LC-MS analyses were carried out on Dionex Ultimate 3000 HPLC system interfaced with an LTQ XL (Thermo Scientific, Waltham, MA, USA) ion trap mass spectrometer. For the oligonucleotides, a Kinetex C18 (150 × 3 mm, 3.6 µm) column was used and the eluents were A: 100 mM HFIP, 1.55 mM DIPEA in H_2_O and B: 100 mM HFIP and 1.55 mM DIPEA in ACN using a gradient from 0–80% B over 12 min at a 0.4 mL/min flow rate. The spectra were recorded at tnegative ion mode. The mass spectra for peptides were recorded in positive ionization mode using eluents: A: H_2_O with 0.1% HCOOH and B: ACN with 0.1% HCOOH on a Phenomenex Peptide XB-C18 column, using a gradient 5–80% B over 25 min at 0.7 mL/min flow rate.

The oligonucleotide and conjugate LC-UV analysis and purification were carried out using a Shimadzu LC-20 system. The eluents were: A: H_2_O with 0.1 M TEAAc and B: ACN: A eluent 8:2 (TEAAc pH 7). The conjugates were purified using an Aviator C18 (150 × 4.6 mm, 3.6 µm) column with a gradient of 5–30% B over 15 min using a 1 mL/min flow rate.

The preparative HPLC for peptides was carried out on a JASCO PU-4180 system equipped with a diode array detector (MD-4015) and an automatic fraction collector (Advantec, CHF122SC). The fraction collection was monitored and programmed using ChromNAV software Version 2.02.08.

The oligonucleotide concentration was determined using a NanoDrop^TM^ One instrument (Thermo Scientific) using the calculated extinction coefficients.

### 2.2. Synthesis of Thiol-Modified Monomer

3-(*tert*-Butyldisulfanyl)propanoic acid (**7**)

First, 2.94 g (13.7 mmol) of 3-(2-pyridyldisulfanyl)propanoic acid **6** was dissolved in 50 mL of 1,2-dichloroethane, then 3.1 mL (2.47 g, 2 equiv.) of 2-methylpropane-2-thiol and 5 drops of acetic acid were added. The reaction mixture was stirred at 55 °C overnight. After evaporation, the crude product was purified on silica using 20% (*v*/*v*) EtOAc in hexanes. The yield was 1.97 g (74%) of colorless oil, R_f_: 0.80 (hexanes-EtOAc-acid 50:50:2).

^1^H NMR (Chloroform-*d*) δ 2.92 (t, *J* = 7.3 Hz, 2H, CH_2_CH_2_), 2.78 (t, *J* = 7.3 Hz, 2H, CH_2_CH_2_), and 1.34 (s, 9H, C(CH_3_)_3_).

^13^C NMR (Chloroform-*d*) δ 178.02 (COOH), 48.17 (**C**(CH_3_)_3_), 34.54 (CH_2_CH_2_), 34.17 (CH_2_CH_2_), and 30.09 (C(**C**H_3_)_3_).

The ESI-MS (negative ion mode) was 193.0 [M-H]^−^.

5-(Azidomethyl)-3’,5’-bis-*O*-[*tert*-butyl(dimethyl)silyl]-2’-deoxyuridine (**4**)

A modified procedure by Gubu et al. [38] has been applied as described: 4.7 g (10 mmol) of nucleoside **2** was dissolved in 100 mL of anhydrous CCl_4_, then 3.54 g (20 mmol, 2 equiv) of NBS and 0.82 g (5 mmol, 0.5 equiv) of AIBN was added. The reaction mixture was stirred at 70 °C under Ar atmosphere for 3 h. The TLC showed incomplete conversion of the starting material, therefore another 0.89 g (5 mmol, 0.5 equiv.) of NBS and 0.82 g (5 mmol, 0.5 equiv) of AIBN were added. The mixture was stirred for another 3 h when the TLC showed an almost complete conversion. The excess of NBS and the succinimide were filtered off and 100 mL of anhydrous DMF was added to the solution. After evaporation of the CCl_4_, 1.63 g (25 mmol, 2.5 equiv.) of NaN_3_ was added and the mixture was stirred at room temperature overnight. The DMF was evaporated and the residue was dissolved in EtOAc (100 mL) and washed twice with 100 mL of aqueous NaHSO_4_ (1%). The organic phase was dried on Na_2_SO_4_ and after the removal of the solvent under reduced pressure, the crude product was chromatographed on silica gel using 10–20% (*v*/*v*) of EtOAc in hexanes to obtain an oily residue, yield: 3.07 g (60%).

^1^H NMR (Chloroform-*d*) δ 7.69 (s, 1H, 6-CH), 6.22 (dd, *J* = 7.8, 5.9 Hz, 1H, 1′-CH), 4.37 (dt, *J* = 5.6, 2.6 Hz, 1H, 3′-CH), 3.91 (q, *J* = 3.4 Hz, 1H, 4′-CH), 3.76 (tdd, *J* = 11.2, 6.8, 3.7 Hz, 2H, 5′-CH_2_), 3.70 (CH_2_NH_2_, d, *J* = 4.3 Hz, 2H), 2.27 (ddd, *J* = 13.3, 5.9, 2.7 Hz, 1H, 2′-CH_2_), 2.10–2.02 (m, 1H, 2′-CH_2_), 0.89 (d, *J* = 7.8 Hz, 18H, 2x(CH_3_)_3_C), and 0.13–0.02 (m, 12H, 4xCH_3_-Si).

^13^C NMR (Chloroform-*d*) δ 164.34 (4-C), 150.19 (2-C), 139.30 (6-CH), 110.02 (5-C), 88.09 (4′-C), 85.53 (1′-CH), 72.48 (3′-CH), 63.23 (5′-CH_2_), 41.06 (2′-CH_2_), 38.17 (CH_2_NH_2_), 26.05 ((**C**H_3_)_3_C), 25.89 ((**C**H_3_)_3_C), 18.50 ((CH_3_)_3_**C**), 18.09 ((CH_3_)_3_**C**), −4.49 (CH_3_Si), −4.70 (CH_3_Si), −5.17 (CH_3_Si), and −5.27 (CH_3_Si).

The HRMS (ESI^+^), calculated for C_22_H_44_N_3_O_5_Si_2_^+^ 486.2814, measured 486.2811 (ΔM: −0.6 ppm).

5-(Aminomethyl)-3′,5′-bis-*O*-[*tert*-butyl(dimethyl)silyl]-2′-deoxyuridine (**5**)

First, 3.07 g (6 mmol) of azide **4** was dissolved in 60 mL of THF, then 3.14 g (12 mmol, 2 equiv.) of Ph_3_P and 2.16 mL (120 mmol, 20 equiv.) water was added. The reaction mixture was kept at 60 °C for 3 h, then the solvent was evaporated and the residue was column chromatographed on silica using 5–15% (*v*/*v*) MeOH in EtOAc. The yield was 2.3 g, white foam (78%).

^1^H NMR (Chloroform-*d*) δ 9.56 (s, 1H, CONH), 7.68 (s, 1H, 6-CH), 6.24 (dd, *J* = 7.8, 5.8 Hz, 1H, 1′-CH), 4.38 (dt, *J* = 5.6, 2.6 Hz, 1H, 3′-CH), 4.08 (d, *J* = 5.8 Hz, 2H, 5-CH_2_), 3.93 (q, *J* = 3.6 Hz, 1H, 4′-CH), 3.77 (t, *J* = 3.6 Hz, 2H, 5′-CH_2_), 2.93–2.88 (m, 2H, CH_2_CH_2_), 2.56–2.47 (m, 2H, CH_2_CH_2_), 2.27 (ddd, *J* = 13.2, 5.8, 2.6 Hz, 1H, 2′-CH_2_), 2.08–2.04 (m, 1H, 2′-CH_2_), 1.30 (s, 9H, (CH_3_)_3_CS), 0.89 (d, *J* = 11.5 Hz, 18H, 2×(CH_3_)_3_CSi), and 0.09 (dd, *J* = 16.9, 1.9 Hz, 12H, 4xCH_3_Si).

^13^C NMR (Chloroform-*d*) δ 170.96 (CONH), 164.09 (4-C), 150.23 (2-C), 138.90 (6-CH), 111.15 (5-C), 88.16 (4′-CH), 85.61 (1′-CH), 72.52 (3′-CH), 63.26 (5′-CH), 48.02 ((CH_3_)_3_**C**S), 41.16 (2′-CH_2_), 36.83 (5-CH_2_), 36.17 (CH_2_CH_2_), 35.78 (CH_2_CH_2_), 30.05 ((**C**H_3_)_3_CS), 26.08 ((CH_3_)_3_CSi), 25.86 ((**C**H_3_)_3_CSi), 18.52, 18.09 (CH_3_)_3_**C**Si), −4.54 (CH_3_Si), −4.71 (CH_3_Si), −5.17 (CH_3_Si), and −5.31 (CH_3_Si).

HRMS (ESI^+^), calculated for C_29_H_56_N_3_O_6_S_2_Si_2_^+^ 662.3144, measured 662.3140 (ΔM: −0.6 ppm).

3′,5′-Bis-*O*-[*tert*-butyl(dimethyl)silyl]-5-{[3-(*tert*-butyldisulfanyl)propanamido]-methyl}-2′-deoxyuridine (**8**)

First, 1.46 g (7.6 mmol) of 3-(*tert*-butyldisulfanyl)propanoic acid (**7**), 1.16 g (7.6 mmol) of HOBt, 1.36 mL (8 mmol) of DIPEA, and 2.84 g (7.6 mmol) HBTU were dissolved in 40 mL of anhydrous DMF. In 10 min, 3.04 g (6.2 mmol, 0.8 equiv.) of amine **5** was dissolved in 30 mL of anhydrous DMF and was mixed with the activated carboxylic acid **7** solution; the mixture was stirred for 2 h. The TLC did not show any ninhydrin-active spots; therefore, the reaction mixture was evaporated, dissolved in EtOAc (100 mL), washed twice with 1% NaHSO_4_ solution (2 × 100 mL), dried over Na_2_SO_4_, and evaporated. The crude oily material was column chromatographed using 30–40% (*v*/*v*) EtOAc in hexanes eluent. The yield was 3.0 g (73%) pale yellow oil.

^1^H NMR (500 MHz, Chloroform-*d*) δ 9.56 (s, 1H, CONH), 7.68 (s, 1H, 6-CH), 6.24 (dd, *J* = 7.8, 5.8 Hz, 1H, 1’-CH), 4.38 (dt, *J* = 5.6, 2.6 Hz, 1H, 3’-CH), 4.08 (d, *J* = 5.8 Hz, 2H, 5-CH_2_), 3.93 (q, *J* = 3.6 Hz, 1H, 4’-CH), 3.77 (t, *J* = 3.6 Hz, 2H, 5’-CH_2_), 2.93–2.88 (m, 2H, CH_2_CH_2_), 2.56 – 2.47 (m, 2H, CH_2_CH_2_), 2.27 (ddd, *J* = 13.2, 5.8, 2.6 Hz, 1H, 2’-CH_2_), 2.08–2.04 (m, 1H, 2’-CH_2_), 1.30 (s, 9H, (CH_3_)_3_CS), 0.89 (d, *J* = 11.5 Hz, 18H, 2×(CH_3_)_3_CSi), 0.09 (dd, *J* = 16.9, 1.9 Hz, 12H, 4×CH_3_Si).

^13^C NMR (126 MHz, CDCl_3_) δ 170.96 (CONH), 164.09 (4-C), 150.23 (2-C), 138.90 (6-CH), 111.15 (5-C), 88.16 (4′-CH), 85.61 (1’-CH), 72.52 (3′-CH), 63.26 (5′-CH), 48.02 ((CH_3_)_3_**C**S), 41.16 (2′-CH_2_), 36.83 (5-CH_2_), 36.17 (CH_2_CH_2_), 35.78 (CH_2_CH_2_), 30.05 ((**C**H_3_)_3_CS), 26.08 ((CH_3_)_3_CSi), 25.86 ((**C**H_3_)_3_CSi), 18.52, 18.09 (CH_3_)_3_**C**Si), −4.54 (CH_3_Si), −4.71 (CH_3_Si), −5.17 (CH_3_Si), −5.31 (CH_3_Si).

HRMS (ESI^+^): Calculated for C_29_H_56_N_3_O_6_S_2_Si_2_^+^ 662.3144, measured 662.3140 (ΔM: −0.6 ppm).

5-{[3-(*tert*-Butyldisulfanyl)propanamido]methyl}-2’-deoxyuridine (**9**)

First, 1.46 g (2.21 mmol) of nucleoside **8** was dissolved in 20 mL of THF and 2.8 g (8.84 mmol, 2 equiv.) of tetrabutylammonium fluoride trihydrate was added. The mixture was stirred at 60 °C for 2 h when the TLC showed complete deprotection. After evaporation, the crude product was chromatographed on silica gel using 2–10% (*v*/*v*) methanol in EtOAc. The yield was 0.80 g (84%) of white foam.

^1^H NMR (Methanol-*d*_4_) δ 7.91 (s, 1H, 6-CH), 6.26 (t, *J* = 6.7 Hz, 1H, 1′-CH), 4.39 (dt, *J* = 6.6, 3.4 Hz, 1H, 3′-CH), 4.04 (s, 2H, C**H**_2_NH), 3.92 (q, *J* = 3.8 Hz, 1H, 4′-CH_2_), 3.83–3.68 (m, 2H, 5′-CH_2_), 2.94 (t, *J* = 7.2 Hz, 2H, CH_2_CH_2_), 2.58 (t, *J* = 7.2 Hz, 2H, CH_2_CH_2_), 2.31–2.18 (m, 2H, 2′-CH_2_), and 1.32 (s, 9H, (CH_3_)_3_).

^13^C NMR (Methanol-*d*_4_) δ 173.92 (CONH), 165.18 (4-C), 152.11 (2-C), 140.12 (6-CH), 112.15 (5-C), 89.02 (4′-CH), 86.63 (1′-CH), 72.30 (3′-CH), 63.04 (5′-CH_2_), 48.53 ((CH_3_)_3_**C**), 41.28 (2′-CH_2_), 37.07 (CONHC**H**_2_), 37.05 (CH_2_CH_2_), 36.67 (CH_2_CH_2_), 30.34 ((**C**H_3_)_3_C), and 30.30 ((**C**H_3_)_3_C).

The HRMS (ESI^+^), calculated for C_17_H_28_N_3_O_6_S_2_^+^ 434.1414, measured 434.1408 (ΔM: −1.4 ppm).

5′-*O*-[Bis(4-methoxyphenyl)(phenyl)methyl]-5-{[3-(*tert*-butyldisulfanyl)-propanamido]methyl}-2′-deoxyuridine (**10**)

First, 0.8 g (1.85 mmol) of nucleoside **9** was dissolved in 15 mL of pyridine and 0.94 g (2.77 mmol, 1.5 equiv.) of 4,4′-dimethoxytrityl chloride was added. The reaction mixture was stirred at room temperature until it became homogeneous then left at room temperature overnight. The TLC showed almost full conversion; therefore, the pyridine was evaporated and the residue was dissolved in EtOAc (80 mL) and washed three times with 80 mL of saturated sodium bicarbonate solution. After drying the organic phase on Na_2_SO_4_, the crude product was evaporated and column chromatographed on silica using 50–0% (*v*/*v*) hexanes in EtOAc-triethylamine 98:2 (*v*/*v*) eluent system. The yield was 0.99 g (73%) of white foam.

^1^H NMR (Methanol-*d*_4_) δ 7.74 (s, 1H, 6-H), 7.52–7.12 (m, 9H, aromatic CHs), 6.92–6.80 (m, 4H, aromatic CHs), 6.27 (t, *J* = 6.7 Hz, 1H, 1′-CH), 4.44 (dt, *J* = 8.5, 4.2 Hz, 1H, 3′-CH), 4.00 (q, *J* = 3.9 Hz, 1H, 4′-CH), 3.78 (s, 6H, 2xCH_3_O), 3.66 (q, *J* = 14.6 Hz, 2H, 5′-CH_2_), 3.37 (d, *J* = 3.6 Hz, 2H, CONHC**H**_2_), 2.84 (t, *J* = 7.2 Hz, 2H, CH_2_CH_2_), 2.41 (td, *J* = 7.3, 3.5 Hz, 2H, CH_2_CH_2_), 2.35–2.28 (m, 2H, 2′-CH_2_), and 1.30 (s, 9H (CH_3_)_3_).

^13^C NMR (Methanol-*d*_4_) δ 173.44 (CONH), 165.08 (4-C), 160.22 (CH_3_O**C**), 152.08 (2-C), 146.13 (DMTr-C_q_), 140.32 (6-CH), 137.12 (DMTr-C_q_), 136.95 (DMT-CH), 131.39 (DMT-CH), 131.36 (DMT-CH), 129.41 (DMT-CH), 128.93 (DMT-CH), 128.02 (DMT-CH), 114.26 (DMT-CH), 111.91 (5-C), 87.94 (DMTr-C_q_), 87.65 (4′-CH), 86.47 (1′-CH), 72.52 (3′-CH), 64.99 (5′-CH_2_), 55.77 (CH_3_O), 48.55 ((CH_3_)_3_**C**), 41.19 (2′-CH_2_), 37.32 (CH_2_CH_2_), 37.03 (CH_2_CH_2_), 36.56 (CONH**C**H_2_), and 30.30 ((CH_3_)_3_).

HRMS (ESI^+^), calculated for C_38_H_45_N_3_O_8_S_2_Li^+^ 742.2803, measured 742.2790 (ΔM: −1.8 ppm).

5′-*O*-[Bis(4-methoxyphenyl)(phenyl)methyl]-5-{[3-(*tert*-butyldisulfanyl)-propanamido]methyl}-3′-*O*-{(2-cyanoethoxy)[di(propan-2-yl)amino]phosphanyl}-2′-deoxyuridine (**11**)

First, 0.87 g (1.18 mmol) of nucleoside **10** was dissolved in dry acetonitrile (30 mL) and under argon atmosphere 308 µL (229 mg, 1.77 mmol, and 1.5 equiv.) of *N*,*N*-diisopropylethylamine followed by 329 µL (349 mg, 1.48 mmol, and 1.25 equiv.) of 2-cyanoethyl *N*,*N*-di(propan-2-yl)phosphoramidochloridite were added. The reaction mixture was stirred at room temperature for 30 min when the TLC showed the complete conversion of the starting nucleoside. The reaction mixture was evaporated and the residue was dissolved in a small amount of EtOAc (2–4 mL) and introduced to a short silica column to subject column chromatography. Before the chromatography, the column was washed with 2 column volume of hexanes-EtOAc-triethylamine 50:50:2 (*v*/*v*), then 5 column volume of hexanes-EtOAc 50:50 (*v*/*v*) to remove the excess of triethylamine. The elution was implemented using 25–0% (*v*/*v*) hexanes in EtOAc and the product-containing UV active fractions were evaporated and co-evaporated using acetonitrile (to remove the traces of triethylamine). The yield was 783 mg (71%) of white foam.

^1^H NMR (C_6_D_6_) δ 10.34 (s, 1H, NH), 7.88 (d, *J* = 3.6 Hz, 1H, 6-CH), 7.73–7.66 (m, 2H, DMT CHs), 7.56 (dt, *J* = 9.2, 3.1 Hz, 4H, DMT CHs), 7.28 (t, *J* = 7.6 Hz, 2H, DMT CHs), 7.12 (t, *J* = 7.3 Hz, 1H, DMT CH), 6.94–6.87 (m, 4H, DMT CHs), 6.40 (q, *J* = 6.5 Hz, 1H, 1′-CH), 4.71 (dt, *J* = 10.3, 5.0 Hz, 1H, 3′-CH), 4.20 (dt, *J* = 13.4, 4.1 Hz, 1H, 4′-CH), 3.98–3.79 (m, 4H, CONHC**H**_2_ and POCH_2_), 3.66–3.46 (m, 4H, 5′-CH_2_ and CH_2_CN), 3.45–3.42 (m, 6H, OCH_3_), 3.38–3.19 (m, 2H, C**H**(CH_3_)_2_), 2.94 (t, *J* = 7.4 Hz, 2H, CH_2_CH_2_), 2.36 (t, *J* = 7.3 Hz, 2H, CH_2_CH_2_), 2.29 (dt, *J* = 13.9, 7.2 Hz, 1H, 2′-CH_2_), 1.94 (dt, *J* = 12.1, 6.2 Hz, 1H, 2′-CH_2_), 1.20 (d, *J* = 1.8 Hz, 9H, (CH_3_)_3_C), 1.11 (t, *J* = 7.4 Hz, 9H, (CH(C**H**_3_)_2_), and 1.03 (d, *J* = 6.8 Hz, 3H, (CH(C**H**_3_)_2_).

^13^C NMR (C_6_D_6_) δ 170.75/170.73 (CONH), 164.34/164.32 (4-C), 159.35/159.33 (CH_3_O**C**), 150.75/150.67 (2-C), 145.64/145.60 (DMTr-C_q_), 139.28/139.22 (6-CH), 136.29/136.25 (DMTr-C_q_), 130.86/130.83, 130.81/130.79, 128.83/128.81, 128.40/128.35 and 127.26/127.24 (DMTr-CHs), 117.81/117.60 (CN), 113.87 (5-C), 111.95/111.93 (DMTr-CH), 87.25 (DMTr-C_q_), 85.83/85.80, 85.66/85.64 and 85.60/85.55 (4′-C and 1′-C), 73.59/73.45/73.33/73.20 (3′-CH), 63.58/63.51 (5′-CH_2_), 60.09 (**C**H_2_CN), 58.84/58.69 (POCH_2_), 55.00/54.97 (CH_3_O), 47.73 ((CH_3_)_3_C), 43.64/43.60 and 43.54/43.50 (**C**H(CH_3_)_2_), 40.23/39.92 (2′-CH), 36.86 (CONH**C**H_2_), 36.51 and 36.15 (**C**H_2_**C**H_2_), 30.02 ((**C**H_3_)_3_C), and 24.75/24.72/24.70/24.67/24.66/24.64/24.61 (CH(**C**H_3_)_2_).

^31^P NMR (C_6_D_6_) δ were 148.98 and 148.71.

HRMS (ESI^+^), calculated for C_47_H_62_N_5_O_9_PS_2_Li_2_^+^ 942.3881, measured 942.3881 (ΔM: 0 ppm).

### 2.3. Oligonucleotide Synthesis

The synthesis of the DNA oligonucleotides was performed using a K&A H-16 synthesizer (K&A Labs GmbH, Schaafheim, Germany) using standard β-cyanoethyl phosphoramidite chemistry at a nominal scale of 0.2 µmol. The phosphorylation was carried out using solid chemical phosphorylating reagent (CPR II, Bioresearch Technologies, Hoddesdon, UK). The 5′ fluorescein modification was carried out using 6-Carboxyfluoresceinamide phosphoramidite Pro (Primetech ACL, Minsk, Belarus).

The oligonucleotides with *t*-Bu-SS-protected thymidine phosphoramidite **11** were prepared using an ETT coupling reagent, using a 10 min coupling time. The cleavage was performed using concentrated NH_3_ or AMA (ammonium hydroxide/40% aqueous methylamine 1:1 *v*/*v*) for 24 h yielding the *t*-Bu-SS-protected oligonucleotide. The oligonucleotides with S-Bz-Thiol-Modifier C6-dT (Glen Research, Sterling, VA, USA) were prepared using Ac-C and dmf g using DCI as the coupling reagent. Before cleavage, the synthesis column was treated with 20% diethylamine in acetonitrile to cleave the cyanoethyl protecting groups [39] to avoid alkylation of the thiol by acrylonitrile [40,41,42,43]. This was followed by cleavage and deprotection using AMA for 24 h at room temperature, yielding the oligonucleotide with the free thiol group. The oligonucleotides were analyzed and purified by reverse phase HPLC or LC-MS.

### 2.4. Peptide Synthesis, Purification, and Analysis

The peptides were synthesized using a microwave-assisted automated peptide synthesizer (Liberty Blue, CEM, Matthews, NC, USA). The peptides were synthesized on a 0.1 mmol scale on Tentagel R RAM resin (resin loading 0.19 mmol/g, Iris Biotech, Marktredwitz, Germany). The couplings were performed using 5 equivalent amino acid excesses DIC and Oxyma as coupling reagents, dissolved in DMF. The required amounts were calculated using the built-in reagent calculator of Liberty Blue. All the amino acids were double coupled using high swelling (HS) Liberty Blue methods. The deprotection solution contained 10% (*w*/*v*) piperazine dissolved in 10% absolute ethanol/NMP mixture. The cleavage and final deprotection were carried out using TFA/TIS/H_2_O 95/2.5/2.5 (*v*/*v*) mixture for 3 h, stirring at room temperature. The TFA was evaporated and crude peptides were precipitated in ice-cold ether. The precipitate was re-dissolved in AcOH and H_2_O and freeze-dried. The peptides were purified using RP-HPLC on Phenomenex Luna C18 (100 A, 250 × 10 mm) semi-preparative column using eluents A: H_2_O/0.1% TFA and B: ACN/0.1% TFA.

### 2.5. Preparation of Conjugates in Solution Phase

The *t-*Bu-SS protection group was removed using TCEP in solution pH 7 and the oligonucleotide was purified using RP-HPLC. The maleimide functionalized peptides were dissolved in DMSO at a 5 mM concentration. The thiol-functionalized oligonucleotides were dissolved in water at a 200 µM concentration. To prevent disulfide formation of the thiol functionalized oligonucleotides, 2 equivalents of TCEP were added to the stock solution and left for 2 h at room temperature to reduce the potential disulfide bonds. The maleimide thiol ligation was performed from this stock directly at a final concentration of 100 µM oligonucleotide using 5 equivalent peptides (0.5 mM) in 50 mM Tris (pH 7) overnight at room temperature under nitrogen atmosphere. The reaction was followed by LCMS. The conjugates were purified using RP-HPLC.

### 2.6. Preparation of Conjugates on Solid Phase

The CPG-bound oligonucleotides were treated with 100 mM TCEP pH 7 for 3 h and the TCEP solution was changed 3 times. The CPGs were washed with ACN and H_2_O and then reacted with 3-maleimidopropionic acid 10 mM in 50 mM Tris, pH 7.2 20% DMF, overnight. The cleavage was performed using concentrated ammonia, 24 h, at room temperature. The crude product was analyzed using LCMS.

### 2.7. Templated Ligation

The templated ligation of the conjugates was carried out using T4-ligase (Thermo Fischer). The samples contained 1 µL 10× T4-ligase buffer, 1 µL 10 µM template, 0.6 µL 50 µM 5′ phosphorylated 10mer oligonucleotides or peptide oligonucleotide conjugates, 0.75 µL 40 µM extension sequences (O4 and Flu-O5), and 0.5 µL 5 U/µL ligase. The reaction mixtures were diluted with sterilized distilled water to 10 µL. The ligation was carried out in 4 consecutive cycles of 15 min ligation at room temperature, 1 min incubation at 90 °C followed by cooling down to room temperature, and a new aliquot of ligase addition. Before PAGE analysis, 10 µL 2× denaturing DNA dye (14 M Urea, 10× TBE, 0.01% bromophenol blue) was added to the mixtures.

### 2.8. Denaturing DNA Gel Electrophoresis

The acrylamide gels were run on a Biorad MiniProtean system using handcast gels. The gel imaging was performed using a ChemiDoc^TM^ gel documentation system (Biorad, Hercules, CA, USA). A 1 mm thick, 15% acrylamide/8 M urea/TBE gel was cast manually using 5% acrylamide/2.15 M urea/TBE as stacking gel. The gel was run in a 60 °C TBE buffer. To the 10 µL ligation mixtures 10 µL at 60 °C 2× denaturing DNA dye was added, of which 10 µL was loaded onto the gel, followed by running the gel at 110 V for 10 min then 150 V for the rest of the run.

### 2.9. Stability of Thiosuccinimide Bond under Ligation Conditions

The maleimide functionalized peptides were reacted with BME to form the thiosuccinimide product. The peptides were dissolved in DMSO at 5 mM concentration; to 20 µL of this solution, 160 µL water and 20 µL 100 mM BME solution were added. The reactions were followed by LC-MS. After full conversion, the sample was freeze-dried. The freeze-dried sample was redissolved in water and 10× T4 ligation buffer (Thermo Scientific) was added. The sample was heated to 90 °C for 5 min then cooled to room temperature and analyzed using LCMS.

## 3. Results and Discussion

### 3.1. Synthesis of Thiol-Containing Thymidine Nucleoside and Its Incorporation into Oligonucleotide Sequences

To prepare the thiol-modified phosphoramidite, we set out to derivatize the fifth position of a pyrimidine nucleobase with thiol for which a common method is Sonogashira cross coupling [44,45]. In contrast to claims in the literature [44,46], the reactions we attempted to reproduce to create an *N*-acyl-protected 5-propargylamino-modified 2’-deoxyuridine nucleoside provided us with very poor yields (<10%) or almost no products even when we tried to optimize the conditions (solvents, orders of adding the reagents, complex forming bases, catalyst, alternative anhydrous solvents, argon atmosphere, fresh catalyst, etc.). This indicated that the three-carbon length of the acyl-protected propargylamine used here might form a non-suitable chelate complex with the catalyst inhibiting the cross coupling. Therefore, Sonogashira coupling of the pyrimidine nucleosides with acyl-protected alkynylamines is preferred using four- and six-carbon long aminoalkynes [25]. Another possibility to synthesize the required nucleoside monomer containing a *t-*Bu-SS-protected disulfide linker is by using propargyl alcohol for the Sonogashira cross-coupling [47,48,49]. To produce the disulfide, a reaction with di-*tert*-butyl 1-(*tert*-butylthio)-1,2-hydrazinedicarboxylate is necessary, however, this reagent along with the longer aminoalkynes are expensive and have limited availability.

As a result, we started to focus on other solutions and considered using a transition metal-free route. The transition metal-free reactions are becoming more and more popular alternatives in the pharmaceutical industry due to fewer toxicity issues. Moreover, such chemistry can be green and economical [50]. Therefore, we decided to find alternative solutions for the derivatization of 2′-deoxyuridine at the fifth position without disturbing its duplex-forming ability.

**Scheme 1:**
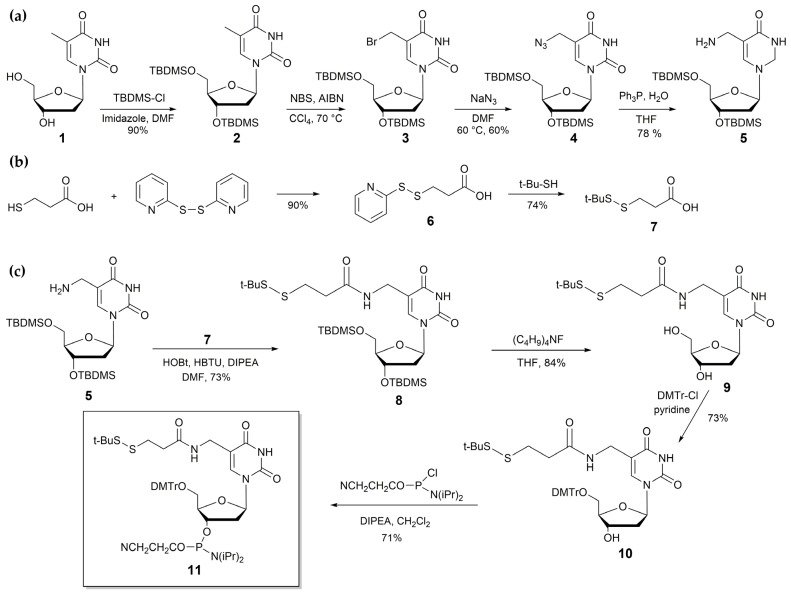
(**a**) Synthesis of amine-modified thymidine at its fifth position (**b**) Synthesis of 3-(2-*tert*-butyldisulfanyl)propanoic acid, and (**c**) Preparation of the *t*-Bu-SS-protected thymidine phosphoramidite. (TBDMS = *tert*-butyldimethylsilyl; DMF = *N*,*N*-dimethylformamide; NBS = *N*-bromosuccinimide; AIBN = azobisisobutyronitrile; DMTr = 4,4′,-dimethoxytrityl; HOBt: 1-hydroxybenzotriazole; HBTU = 3-[bis(dimethylamino)methyliumyl]-3*H*-benzotriazol-1-oxide hexafluorophosphate; DIPEA = *N*,*N*-diisopropyethylamine).

First, we followed the protocol of Gubu et al. [38], which starts from a 3′,5′-diTBDMS protected thymidine **2** (Scheme 1a) and subjects the 5-methyl group to a radical bromination by NBS and AIBN in CCl_4_, then the bromo compound **3** is further reacted with sodium azide in aq. DMF. Unfortunately, this protocol provided several hydrolyzed side-products with a very low yield, therefore we modified the reaction by using anhydrous conditions and, after bromination, the reaction mixture was not worked up as only the solvent was changed to anhydrous DMF; after this, the sodium azide reagent was added. In this way, the azide **4** [38] was isolated in an acceptable yield (60% for the two steps). The azide containing thymidine **4** was reduced by triphenyl phosphine in aq. THF. The resulting amine **5** was coupled with 3-(2-*tert*-butyldisulfanyl)-propanoic acid (**7**) [51], which was prepared according to Navath et al. [37] (Scheme 1b). As a result, the *tert*-butyl-protected disulfide functional group in nucleoside **8** was obtained. The deprotection of silyl groups by a fluoride treatment and the 5′-*O*-DMTr introduction provided the nucleoside **10**, which was transformed into the required nucleoside phosphoramidite **11** using the commercially available phosphitylating agent (Scheme 1c). Although the method we finally optimized contains a few more chemical steps compared to the Sonogashira route, avoiding the transition metal catalyst causes the procedure to be more favorable for its other aspects.

**Figure 2:**
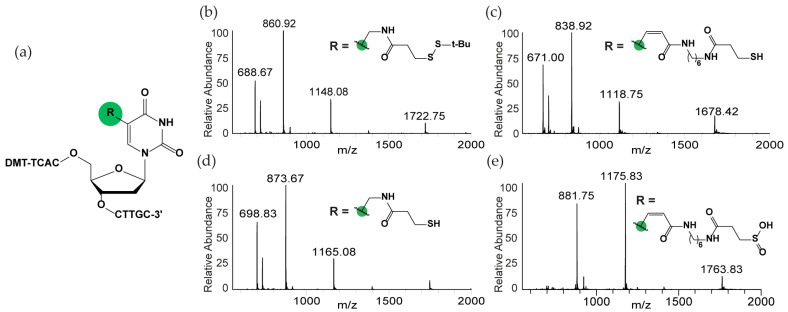
Incorporation of **11** and S-Bz thiol modifier C6 dT to internal oligonucleotide positions. (**a**) Structure of the oligonucleotide with the modified monomer in its fifth position. (**b**) Mass spectrum of the major product of the crude oligonucleotide synthesized using **11** with ETT as the activator, cleavage was performed using AMA for 24 h. DMT-TCACT(*t*-Bu-SS)CTTGC mw: 3447.64, [M-2H]^2−^: 1722.8; [M-3H]^3−^: 1148.1; [M-4H]^4−^: 860.9; and [M-5H]^5−^: 688.7 (**c**) Mass spectrum of the minor product from the same synthesis, DMT-TCACT(SH)CTTGC mw: 3359.47, [M-2H]^2−^: 1678.4; [M-3H]^3−^: 1118.8; [M-4H]^4−^: 838.9; and [M-5H]^5−^: 671.0. (**d**) Mass spectrum of the major product of the crude oligonucleotide synthesized using S-Bz thiol modifier C6 dT using DCI activator, cyanoethyl groups were removed using 20% DEA in ACN then cleavage was performed using AMA 24 h, room temperature. DMT-TCACT(SH)CTTGC mw: 3498.68; [M-2H]^2−^: 1748.3; [M-3H]^3−^: 1165.08; [M-4H]^4−^: 873.7; and [M-5H]^5−^: 698.8. (**e**) Mass spectrum of the minor product from the same cleavage, DMT-TCACT(SOOH)CTTGC mw: 3528.75, [M-2H]^2−^: 1763.8; [M-3H]^3−^: 1175.8; and [M-4H]^4−^: 881.7. LC-MS spectra were recorded in negative ion mode. See also Figure S3 for LCMS chromatograms.

To test the incorporation and the stability of the *t-*Bu-SS protected monomer **11** into oligonucleotide sequences, we first synthesized a dinucleotide using standard β-cyanoethyl phosphoramidite chemistry. The mass spectrum of the crude product after cleavage and deprotection revealed successful coupling, with the *t*-Bu-SS protection group being intact (Figure S1), indicating that the monomer is stable under coupling and deprotection conditions. Next, we incorporated **11** into internal positions of 10mer oligonucleotides (Figure 2a). Using 5-ethylthio-1*H*-tetrazole (ETT) as the activator, we observed the successful incorporation of **11** (Figure 2b), together with the deprotected free thiol as the minor product (Figure 2c and Figure S3). For comparison, the synthesis was carried out with the commercially available S-Bz thiol modifier C6 dT (Figure S2). With this method, the desired product could not be detected and the coupling was only successful when we switched to the 4,5-dicyanoimidazole (DCI) activator. Since the *t-*Bu-SS protection group is more stable under the cleavage conditions, there is no potential risk for side reactions. In contrast, the S-Bz group is completely removed during ammonia treatment and the free thiol is susceptible to degradation and alkylation under these circumstances. To minimize the risks, the 2-cyanoethyl protection groups must be removed first by treating the oligonucleotide with 20% (*v*/*v*) diethylamine (DEA) in ACN [39], then cleaving with ammonium hydroxide/40% aqueous methylamine 1:1 (*v*/*v*) (AMA, instead of ammonia). Following this protocol, we detected the free thiol as the major product (Figure 2d) and as a minor component corresponding to sulfinic acid, formed during the oxidation step (Figure 2e and Figure S3) [31]. Overall, both monomers are suitable for functionalizing the oligonucleotides with thiol mid-sequence but using the *t-*Bu-SS protection group is more advantageous since there is no need to change the coupling reagents during the synthesis and it is less prone to degradation under oxidation and cleavage conditions.

Since our goal was to prepare ligatable oligonucleotide conjugates three 5′ phosphorylated 10mer oligonucleotides were synthesized using **11** in the fifth position (**O1-3(SH)**, Table 1). After cleavage, the *t-*Bu-SS protection group was removed in solution using tris(2-carboxyethyl)phosphine (TCEP) [52]. Following HPLC purification, the thiol-modified oligonucleotides were isolated, which were used in conjugation reactions.

### 3.2. Thiol-Maleimide Conjugation

**Figure 3:**
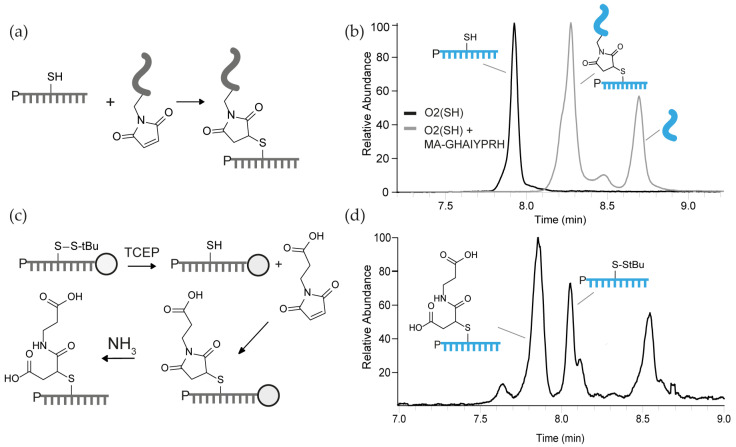
Thiol-maleimide conjugation in solution and on solid phase. (**a**) Reaction scheme for solution phase conjugation using 100 µM oligonucleotide, 200 µM TCEP, and 500 µM peptide, 10% DMSO, overnight room temperature. (**b**) Overlayed LC-MS chromatogram of **O2(SH)** and the crude conjugation product showing full conversion to **O2-GHAIYPRH** conjugate. See Figure S4 for all conjugation reactions. (**c**) Reaction scheme for solid phase conjugation. TCEP treated CPG-bound oligonucleotide was reacted with 3-maleimidopropionic acid (10 mM in 50mM Tris, pH 7.2, 20% DMF) overnight, followed by cleavage using concentrated NH_3_, 24 h. Note that the reaction scheme shows only one possible hydrolysis product, see Figure S6 for an extended reaction scheme. (**d**) LCMS chromatogram of the crude product of solid phase conjugation reaction, after cleavage, see also Figure S6.

We set out to test the conjugation reactions with peptides, which have already been used to prepare oligonucleotide conjugates. These were (i) the transferrin receptor binding **HAIYPRH** (T7) sequence, which is widely utilized to create tumor-targeting delivery systems [53,54]; (ii) **GRGDSP** containing the RGD recognition motif targeting cellular adhesion receptors [55]; and **ASSLNIA**, which has been shown to target muscle cells specifically [56,57]. All the peptides had an N-terminal maleimide functional group that was then reacted with **O1-3(SH)** (Figure 3a and Figure S4). We observed conjugate formation for all three oligonucleotides and the conjugates were successfully purified using RP-HPLC and isolated. We observed differences in conjugation efficiency with **O2-GHAIYPRH** proceeded to full conversion (Figure 3b), whereas in the other two reactions the conjugate formation was less effective (Figure S4). One of the reasons for this could be the presence of TCEP in the reaction mixture, which is a common practice to prevent disulfide formation during bioconjugation reactions. This, however, led to a side reaction between the maleimide moiety of the peptides and TCEP forming the nonproductive ylene by-product [58] (Figure S5). Therefore, the choice of reagent concentrations is crucial for the conjugation reaction; in the future, omitting TCEP entirely could be the best solution to increase conversion.

A further advantage of the *t-*Bu-SS-protected monomer is that the protection group can be selectively removed using reducing reagents while the oligonucleotide is still attached to the solid phase, leaving the other protection groups intact. This opens the possibility to perform conjugation on the solid phase for molecules that can withstand the ammonia cleavage conditions, resulting in the efficient removal of reagents during synthesis and fewer purification steps. To demonstrate this application, the *t-*Bu-SS protection group was removed using TCEP and conjugated to 3-maleimidopropionic acid on the solid phase (Figure 3c,d and Figure S6). We observed efficient conjugate formation using this protocol, with the conjugate as the main product (Figure 3d and Figure S6) separable from the unreacted components and synthesis side-products. During ammonia cleavage, the thiosuccinimide ring hydrolysed and formed a more stable thioether bond, which did not influence the structure of the conjugated moiety.

### 3.3. Templated Ligation of Peptide–Oligonucleotide Conjugates

**Figure 4:**
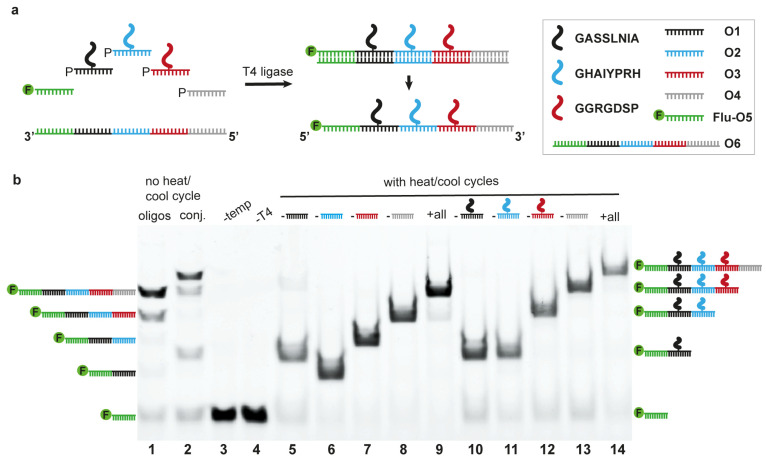
Templated ligation (**a**) Scheme of the ligation process: to the template (**O6**) 5′ phosphorylated oligonucleotides (or peptide–oligonucleotide conjugates) and extension sequences were added with fluorescent labelling on the 5′ end and ligated using T4 ligase. The ligation was carried out using 1 µM template and three equivalent excess oligonucleotides heated to 90 °C and cooled down to room temperature before adding T4 ligase. The heat–cool cycles and ligase additions were repeated three times. (**b**) Denaturing gel electrophoresis of the ligation mixtures with controls. Lanes 1–4: Ligations performed without heat–cool cycles with mixtures containing non-functionalized oligonucleotides (Lane 1) or peptide-oligonucleotide conjugates (Lanes 2–4). For control experiment template, (**O6**, Lane 3) or T4 ligase (Lane 4) was omitted from reaction mixture. Lanes 5–9: Ligation performed using heat–cool cycles with a mixture containing template (**O6**), T4 ligase, oligonucleotides **O1-4** and **Flu-O5**. The following reagents were omitted from the mixture: Lane 5: **O1**; Lane 6: **O2**; Lane 7: **O3**; Lane 8: **O4**; and Lane 9: All reagents present. Lanes 10–14: Ligation performed using heat–cool cycles with a mixture containing template (**O6**), T4 ligase, oligonucleotide conjugates (**O1-GASSLNIA**, **O2-GHAIYPRH**, **O3-GGRGDSP)**, **O4**, and **Flu-O5**. The following reagents were omitted from the mixture: Lane 10: **O1-GASSLNIA**; Lane 11: **O2-GHAIYPRH**; Lane 12: **O3-GGRGDSP**; Lane 14: **O4**; Lane 15: All reagents present. Excluded reagents are indicated on the top of the gel and the full-length and truncated products are shown on the sides of the gel.

With the peptide-functionalized conjugates in hand, our next step was to connect them to a single strand using ligation. T4 ligase can be used to attach short oligonucleotide conjugates to one another, thereby creating DNA strands with multiple functionalities and their arrangement governed by a complementary template sequence [59]. To assemble our peptide–oligonucleotide conjugates, we synthesized a template (**O6**) containing two 15mer extension sequences on both ends (complementary to **O4** and **O5**, Table 1 and Figure 4a). The 3′ reverse complement of the extension sequence was coupled to fluorescein (**Flu-O5)** to ease the detection of the ligated product.

The ligation was first performed with three-fold oligonucleotide excess compared to the template at room temperature, without or with the peptides attached, which led to incomplete ligation products (Figure 4b, Lanes 1–2). To increase the ligation efficiency, the ligation mixture was heated to 90 °C then cooled down to room temperature before the addition of T4 ligase, which was repeated three times. For the control experiments, one of the components was omitted from the ligation mixture. Denaturing PAGE analysis revealed that the ligation proceeded to the desired 60 bp product when all the components were included and the correct truncation product was observed when one of the components was omitted from the reaction (Figure 4b). One exception was when **O1** was omitted from the mixture, where a non-specific 30 bp product formation was formed, but this did not interfere with the ligation when all the other components were present. The presence of peptides did not influence the ligation efficiency; we observed similar yields and product distribution, with product sizes slightly higher than the non-functionalized oligonucleotides, which is expected with the covalently attached peptides. No ligation product could be observed when the template or the ligase was excluded from the reaction, indicating that there are no non-specific interactions.

Since the ligation buffer includes reducing agents and heat is applied, we tested whether the thiosuccinimide bond is stable under these conditions, for which the peptide-maleimides were reacted with beta-mercaptoethanol first and then subjected to the same ligation conditions. Apart from a small amount of hydrolyzed product, no change could be observed (Figure S7), indicating that the conjugates are stable under ligation conditions and that the peptide-functionalized oligonucleotides can readily be prepared via template-directed ligation catalyzed by T4 ligase using standard protocols and reagents.

## 4. Conclusions

In summary, we have shown the applicability of a thiol-modified nucleoside phosphoramidite from its synthesis through conjugation to the preparation of ligatable functionalized oligonucleotides. The monomer presented here was synthesized through a greener, metal-free chemistry and successfully incorporated into oligonucleotide sequences using standard synthesis and cleavage protocols, without the risk of degradation. The thiol-containing oligonucleotides were directly functionalized with peptide-maleimides, without the need for any additional crosslinkers. Furthermore, the selective removal of the *t-*Bu-SS protection group allowed solid-phase conjugation, resulting in fewer purification steps. The prepared peptide–oligonucleotide conjugates were successfully hybridized and ligated on a template resulting in a DNA strand bearing multiple functionalities. Using the presented monomer, it is possible to directly functionalize nucleotides internally and perform conjugations both on solid and in solution phases using thiol reactive reagents. The conjugated moieties do not influence ligase binding, and with the help of a template, virtually endless modifications can be incorporated into spatially defined arrangements, which can be desirable for oligonucleotides used as therapeutics, detections reagents, or in DNA-based display methods.

## Figures and Tables

**Table 1:** Oligonucleotide sequences used for conjugation and ligation.

Name	Sequence (5′–3′) ^1^
O1	p-TGTCTGAACC
O2	p-TCACTCTTGC
O3	p-ACTTTCGCAC
O1(SH)	p-TGTCT(SH)GAACC
O2(SH)	p-TCACT(SH)CTTGC
O3(SH)	p-ACTTT(SH)CGCAC
O4	p-CTTTTATCACGGCCC
Flu-O5	Flu-AGAATGCTGGGCAAT
O6	GGGCCGTGATAAAAGGGTTCAGACAGCAAGAGTGAGTGCGAAAGTATTGCCCAGCATTCT

^1^ T(SH): thiol modified thymidine monomer (**11**); p: 5′ phosphate; Flu: 5′-carboxyfluorescein.

## Data Availability

All data supporting the findings of this study are available within the article and its supplemental information, or from the corresponding author upon reasonable request.

## Supplementary Materials

The following supporting information can be downloaded at: https://www.mdpi.com/article/10.3390/pharmaceutics15010248/s1.
