# Supplementary material for: Improved Metal-Free Approach for the Synthesis of Protected Thiol Containing Thymidine Nucleoside Phosphoramidite and Its Application for the Synthesis of Ligatable Oligonucleotide Conjugates
Source: Pharmaceutics. 2023 Jan 11;15(1):248. doi: 10.3390/pharmaceutics15010248 (PMC9865093; doi:10.3390/pharmaceutics15010248)

Improved metal-free approach for the synthesis of protected thiol containing thymidine nucleoside phosphoramidite and its application for the synthesis of ligatable oligonucleotide conjugates

Zoltán Kupihár [1], Györgyi Ferenc [2], Vencel L. Petrovicz [1], Viktória R. Fáy [1], Lajos Kovács [1], Tamás A. Martinek [1,3,*] and Zsófia Hegedüs [1,*]

[1] Department of Medical Chemistry, University of Szeged, Dom ter 8., H-6720 Szeged, Hungary; kupihar.zoltan@med.u-szeged.hu (Z.K.); petrovicz.vencel.laszlo@med.u-szeged.hu (V.L.P.); fayviki@gmail.com (V.R.F.); kovacs.lajos@med.u-szeged.hu (L.K.)

[2] Institute of Plant Biology, Biological Research Centre, Eötvös Lóránd Research Network, H-6726 Szeged, Hungary; ferenc.gyorgyi@brc.hu

[3] ELKH-SZTE Biomimetic Systems Research Group, Eötvös Loránd Research Network, H-6720 Szeged, Hungary

* Correspondence: martinek.tamas@med.u-szeged.hu (T.A.M.); hegedus.zsofia@med.u-szeged.hu (Z.H.)

Supplementary Materials

Table of Contents

S1 Supplementary Figures 3

S2 Characterization of oligonucleotides and oligonucleotide conjugates 9

S2.1 Characterization of thiol-modified oligonucleotides 9

S2.2 Maleimide functionalized peptides 12

S2.3 LC-MS spectra of pure oligo-peptide conjugates 15

S1 Supplementary Figures and Tables

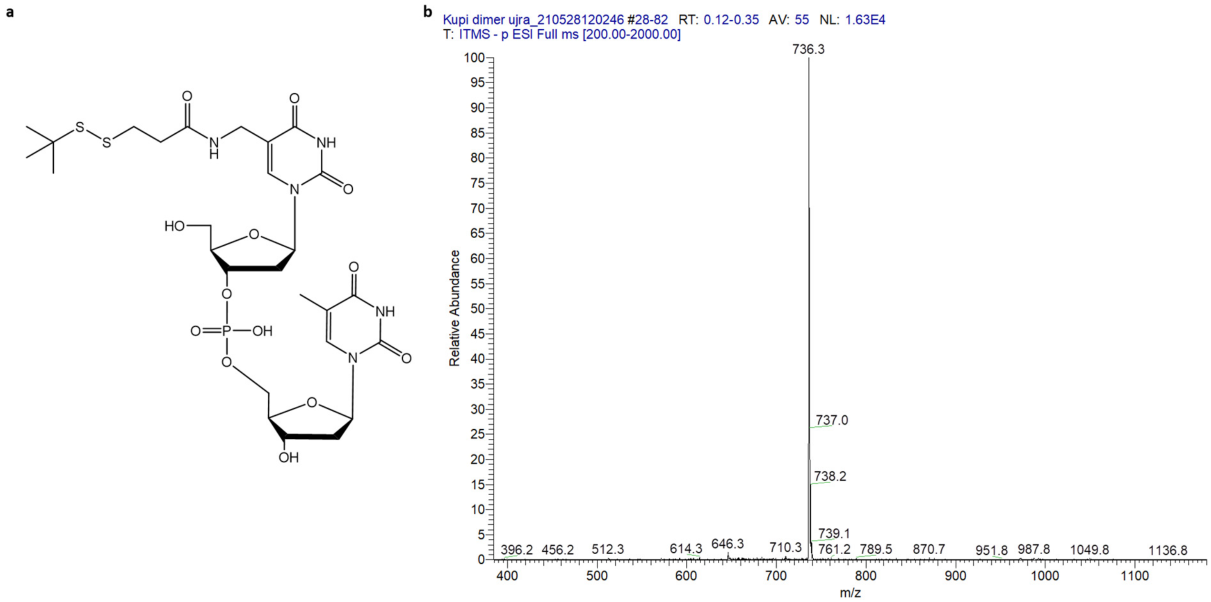

Figure S1. (**a**) Structure of the dinucleotide synthesized using **11**. Synthesis was carried out using standard β-cyanoethyl phosphoramidite chemistry, using ETT as coupling reagent. Cleavage and deprotection was carried out using concentrated ammonia, 24 h, room temperature. (**b**) Mass spectrum of the crude product. T(*t*-Bu-SS)T; exact mass: 737.18. Detected: $[M\text{-}H]^-$: 736.3

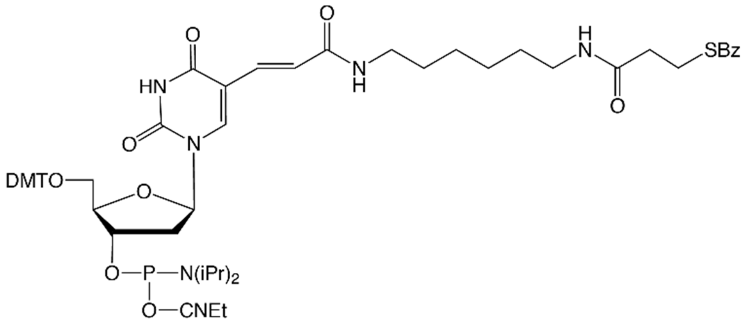

Figure S2. Structure of S-Bz thiol modifier C6 dT (Glen Research)

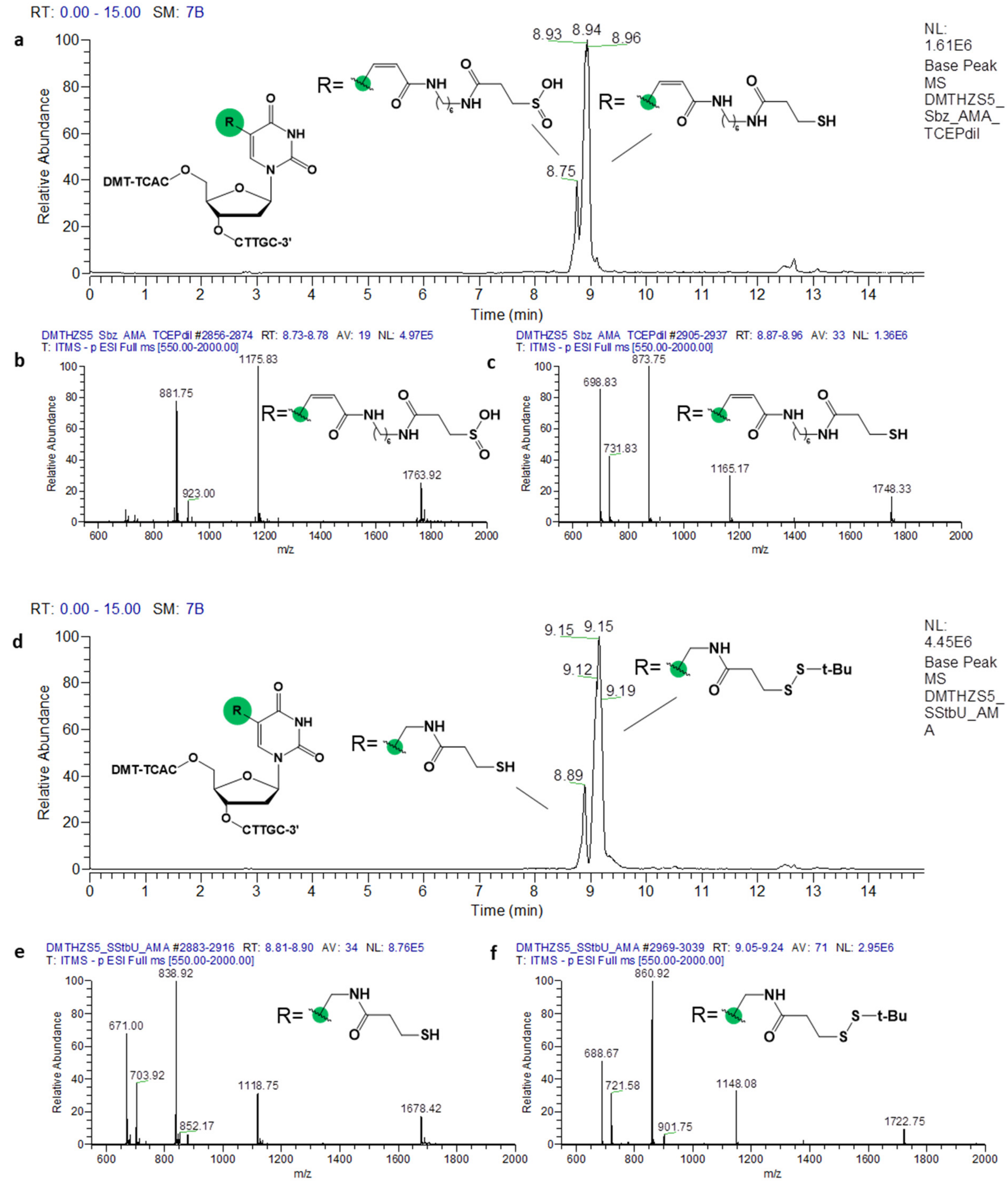

Figure S3. (**a**–**c**) LCMS chromatogram and corresponding mass spectra for the incorporation of **11** to internal oligonucleotide positions using ETT as the activator, cleavage was performed using AMA for 24 h. DMT-TCACT(S-StBu)CTTGC mw: 3447.64, $[M-3H]^{3-}$: 1722.8; $[M-4H]^{4-}$: 1148.1; $[M-5H]^{5-}$: 860.9; $[M-6H]^{6-}$: 688.7; DMT-TCACT(SH)CTTGC mw: 3359.47, $[M-3H]^{3-}$: 1678.4; $[M-4H]^{4-}$: 1118.8; $[M-5H]^{5-}$: 838.9; $[M-6H]^{6-}$: 671.0. (**d**–**f**) LCMS chromatogram and corresponding mass spectra for the incorporation of S-Bz thiol modifier C6 dT to internal oligonucleotide position using DCI activator, cyanoethyl groups were removed using 20% DEA in ACN then cleavage was performed using AMA for 24 h, room temperature. DMT-TCACT(SH)CTTGC mw: 3498.68; $[M-2H]^{2-}$: 1748.3; $[M-3H]^{3-}$: 1165.08; $[M-4H]^{4-}$: 873.7; $[M-5H]^{5-}$: 698.8; DMT-TCACT(SOOH)CTTGC mw: 3528.75, $[M-2H]^{2-}$: 1763.8; $[M-3H]^{3-}$: 1175.8; $[M-4H]^{4-}$: 881.7.

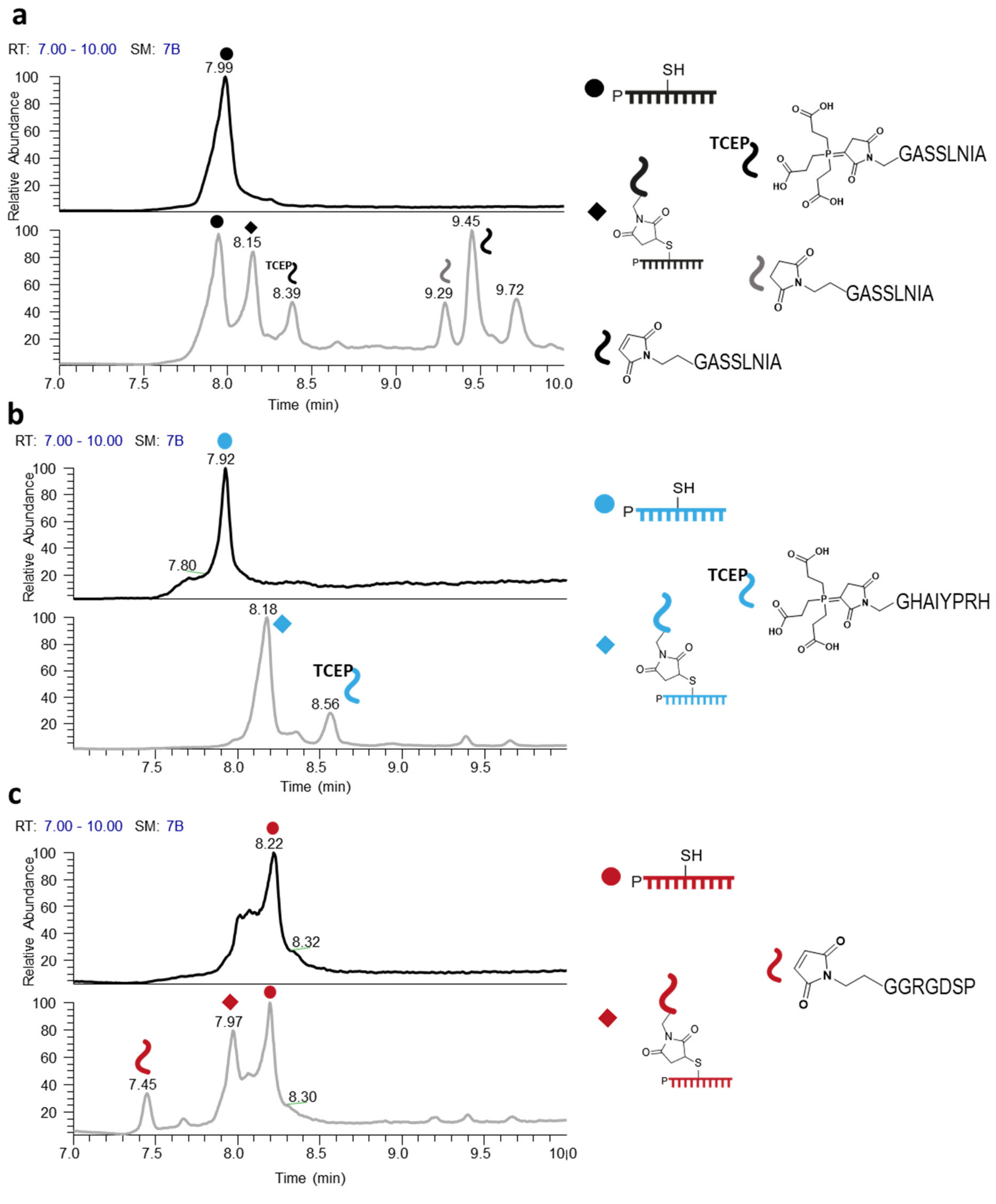

Figure S4. LCMS chromatograms of thiol-modified oligonucleotides (black) and the crude products after conjugation with the maleimide functionalized peptides (grey) for (**a**) **O1(SH)** (**b**) **O2(SH)** and (**c**) **O3(SH)**. Conditions: 500 µM oligonucleotide was treated with 2 equivalents of TCEP at pH7 to prevent disulphide formation, for 2 h then maleimide functionalized peptide was added, final concentrations: 100 µM oligonucleotide, 200 µM TCEP, 500 µM peptide, 10% DMSO, overnight room temperature. Product structures are shown next to the chromatograms: unconjugated oligonucleotide (circle), conjugate (diamond), unreacted peptide (wave) and peptide-TCEP adduct (TCEP).

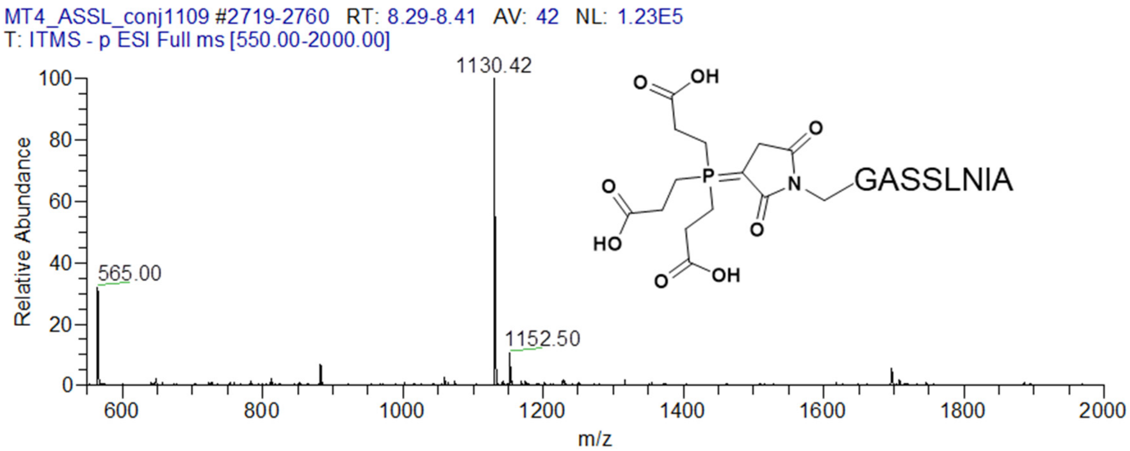

Figure S5. Structure and mass spectrum showing the ylene side-product formed by the reaction between TCEP and maleimide moiety of GASSLNIA during conjugation. Em: 1131.48; $[M\text{-}H]^{-}$: 1130.42; $[M\text{-}2H]^{2-}$: 565.00

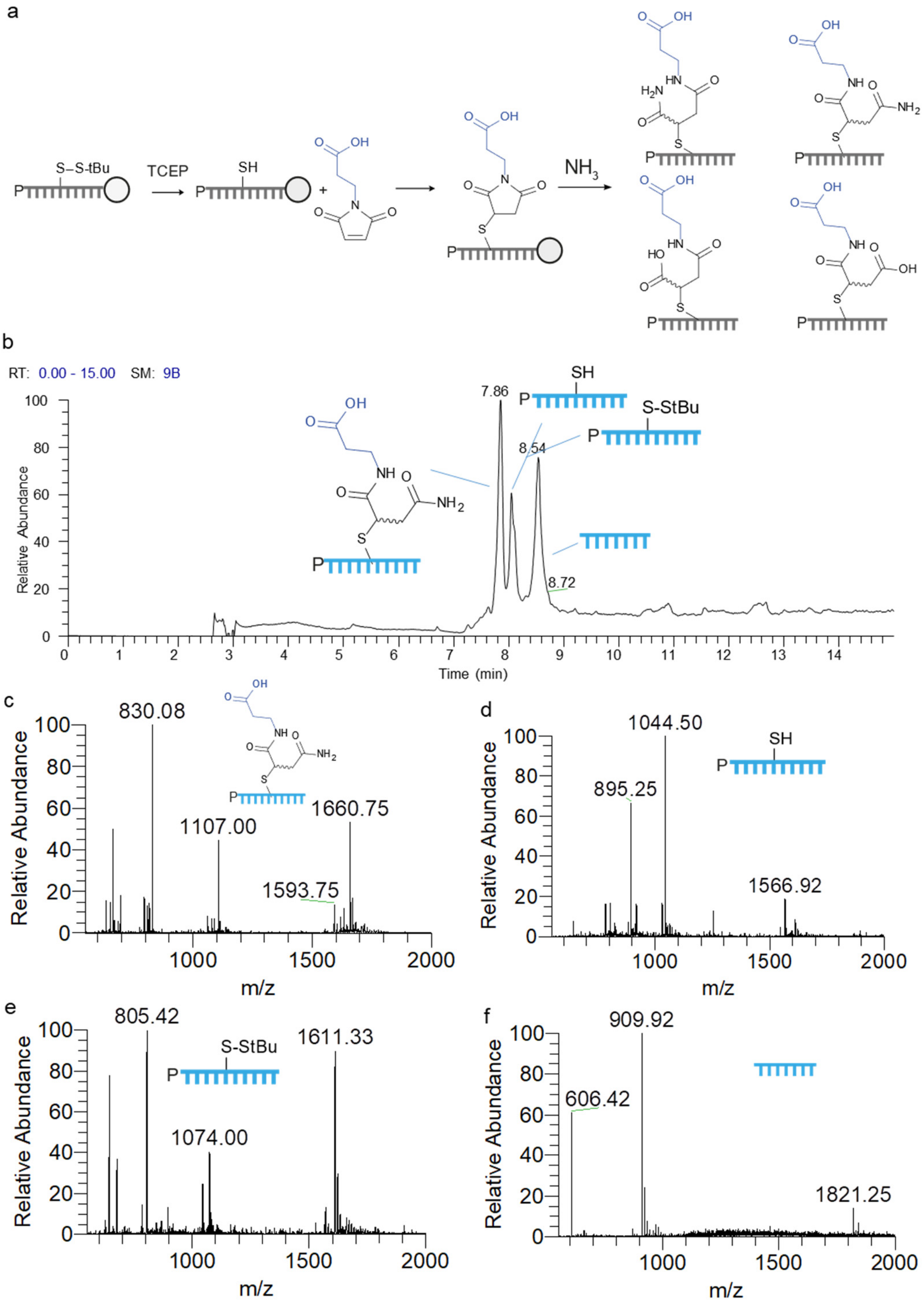

Figure S6. (**a**) Reaction scheme for solid phase conjugation. The CPG-bound oligonucleotide was treated with 100 mM TCEP, pH 7 three times, then 10 mM 3-maleimidopropionic acid was added (50 mM Tris, pH 7.2, 20% DMF) overnight, which was followed by cleavage using concentrated NH_3, 24 h. (**b**) LCMS chromatogram of the crude product and corresponding mass spectra (**c**) O2-MPA hydrolised conjugate MW: 3323.55 (carboxylate) or 3322.55 (amide) $[M-2H]^{2-}$: 1660.75; $[M-3H]^{3-}$: 1107.0; $[M-4H]^{4-}$: 830.08 $[M-5H]^{5-}$: 663.70 (**d**) O2-SH mw: MW: 3137.103; $[M-2H]^{2-}$: 1566.92; $[M-3H]^{3-}$: 1044.50; $[M-4H]^{4-}$: 783.42; (**e**) O2-*t*-Bu-SS MW: 3225.25; $[M-2H]^{2-}$: 1611.33; $[M-3H]^{3-}$: 1074.0 ; $[M-4H]^{4-}$: 805.3; $[M-5H]^{5-}$: 644.05 and (**f**) Mass spectrum corresponding to peak at 8.54 minutes, which we attribute to a shorter synthesis side-product.

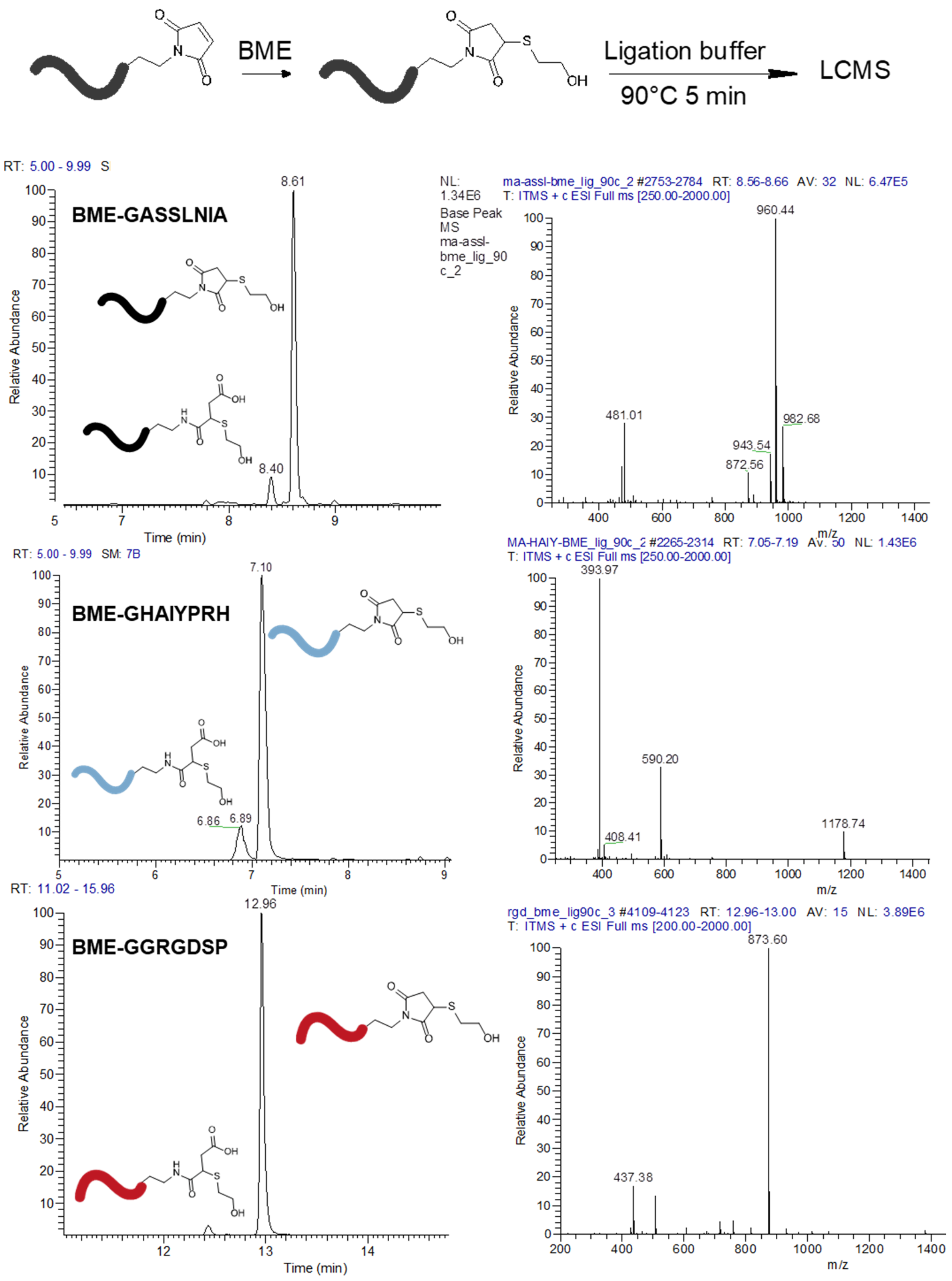

Figure S7. Stability of the peptide thiosuccinimides under ligation conditions. Peptide maleimides were reacted with beta-mercaptoethanol (BME) to form thiosuccinimide derivatives. After the removal of BME, the formed products were added to the ligation buffer and heated to 90°C for five minutes and tested using LCMS. Hydrolysis of the thiosuccinimide ring occurred to some extent under these conditions, but no other change could be detected for the peptides. BME-GASSLNIA Em: 959.44 $[M+H]^+$: 960.44, $[M+2H]^{2+}$: 481.01, $[M+Na]^+$: 982.68, the detected M-17 (m/z: 943.54) and M-88 (m/z: 872.56) peaks are due to fragmentation under MS conditions (see also 3.3) BME-GHAIYPRH Em: 1177.54 $[M+H]^+$: 1178.74, $[M+2H]^{2+}$: 590.20, $[M+3H]^{3+}$: 393.97 BME-GGRGDSP Em: 872.34, $[M+H]^+$: 873.60, $[M+2H]^{2+}$: 437.38

S2 Characterization of oligonucleotides and oligonucleotide conjugates

S2.1 Characterization of thiol-modified oligonucleotides

O1(SH) p-TGTCT(SH)GAACC

MW: 3186.141; $[M-2H]^{2-}$: 1592.83; $[M-3H]^{3-}$: 1061.00; $[M-4H]^{4-}$: 795.67; $[M-5H]^{5-}$: 636.33;

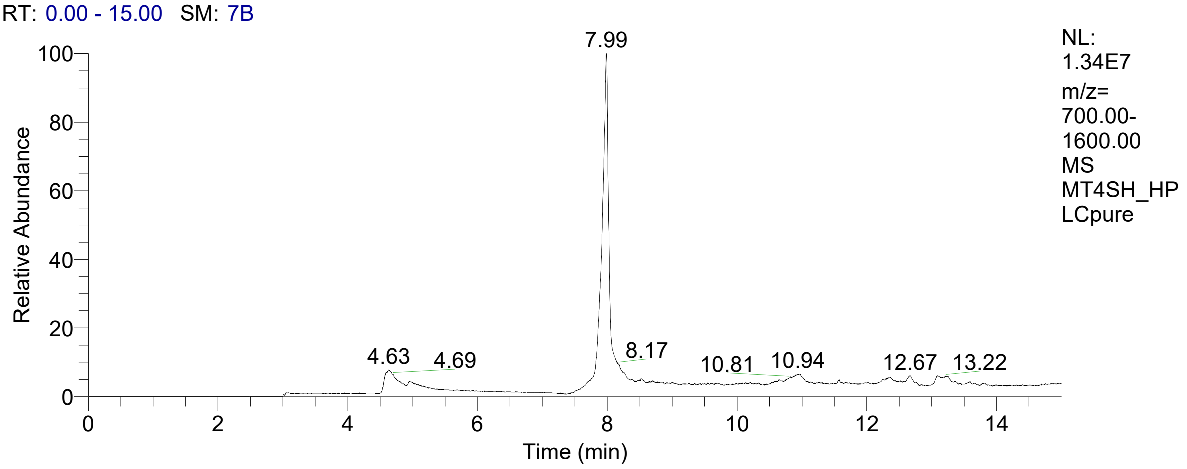

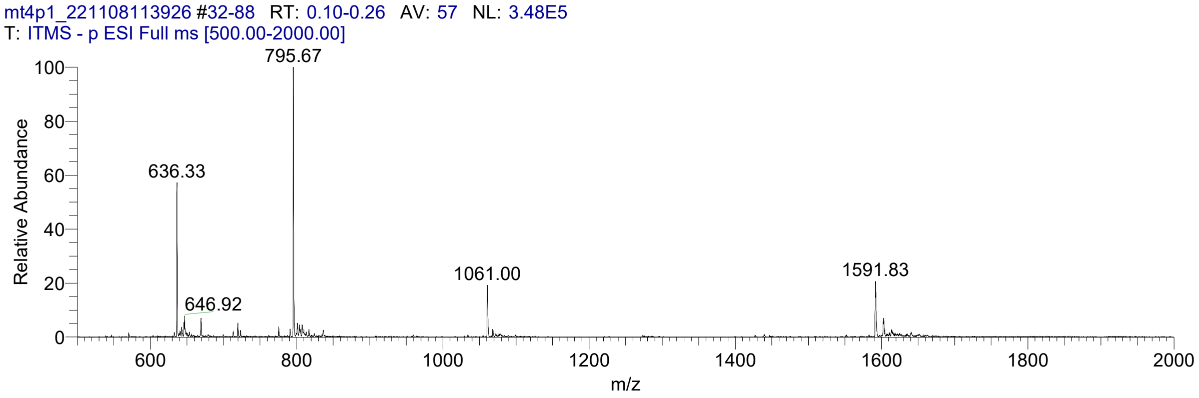

O2(SH) p-TCACT(SH)CTTGC

MW: 3137.103; $[M-2H]^{2-}$: 1567.33; $[M-3H]^{3-}$: 1044.75; $[M-4H]^{4-}$: 783.42; $[M-5H]^{5-}$: 626.58;

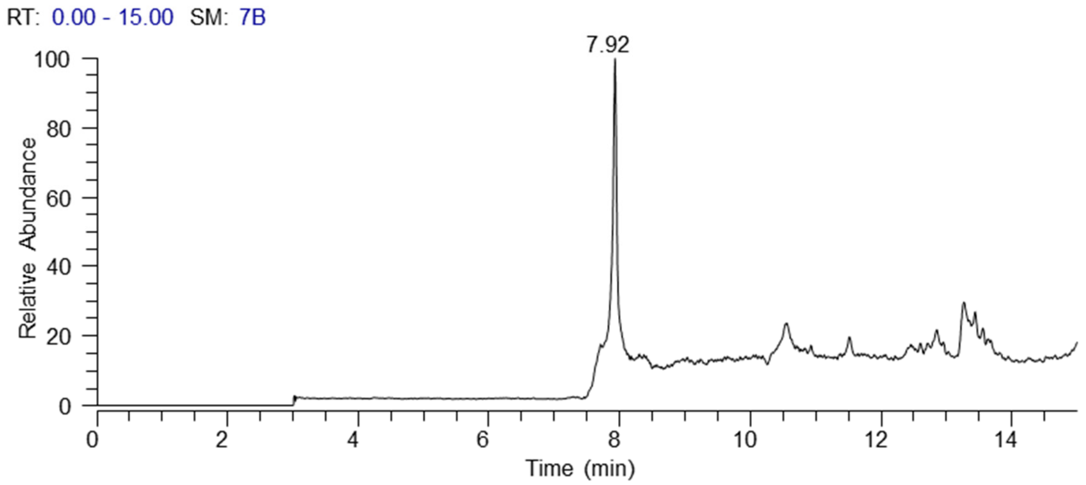

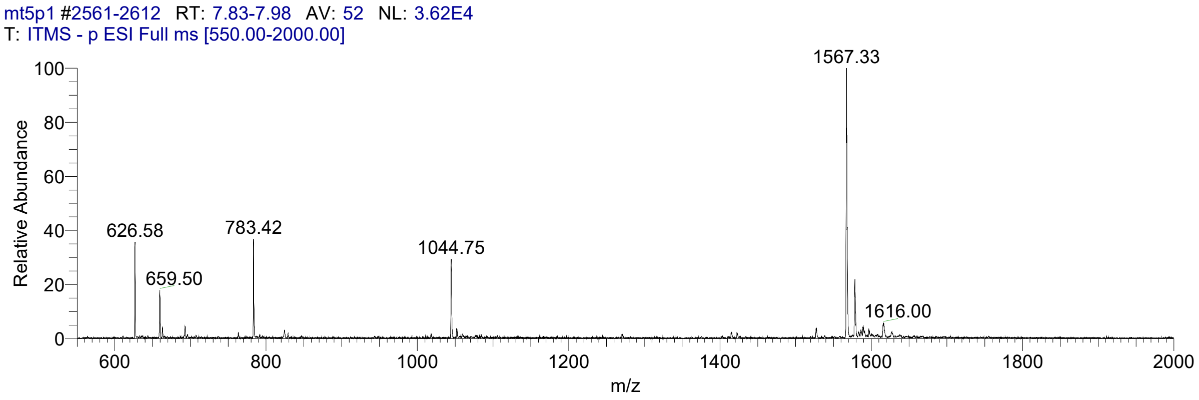

O3(SH) p-ACTTT(SH)CGCAC

MW: 3146.16; $[M-2H]^{2-}$: 1571.92; $[M-3H]^{3-}$: 1047.50; $[M-4H]^{4-}$: 785.58

Dimer MW: 6290.32; $[M-5H]^{5-}$: 1257.17, $[M-7H]^{7-}$: 897.83

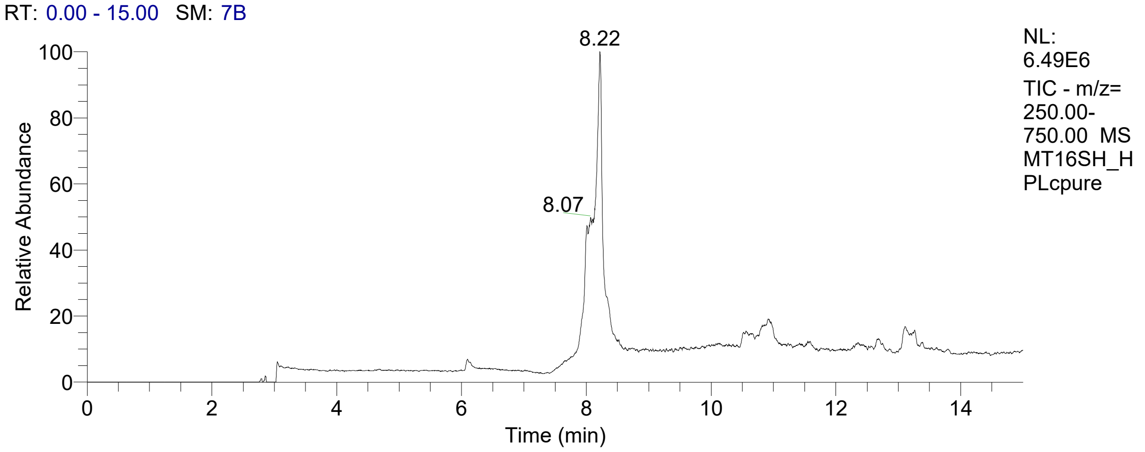

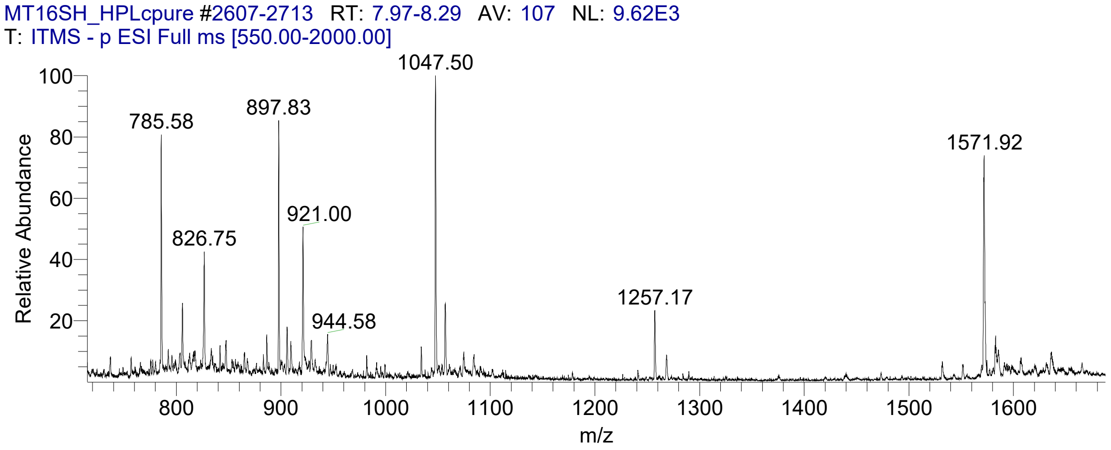

S2.2 Maleimide functionalized peptides

MPA-GASSLNIA

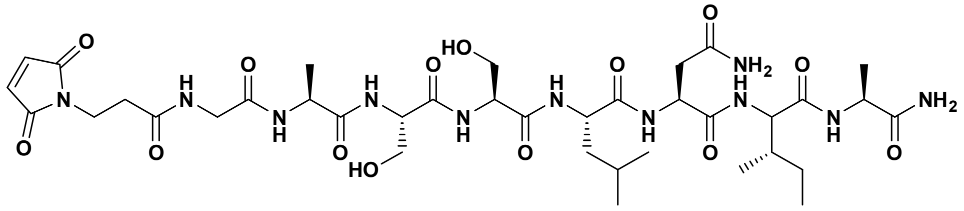

Exact Mass: 881.42

Molecular Weight: 881.94

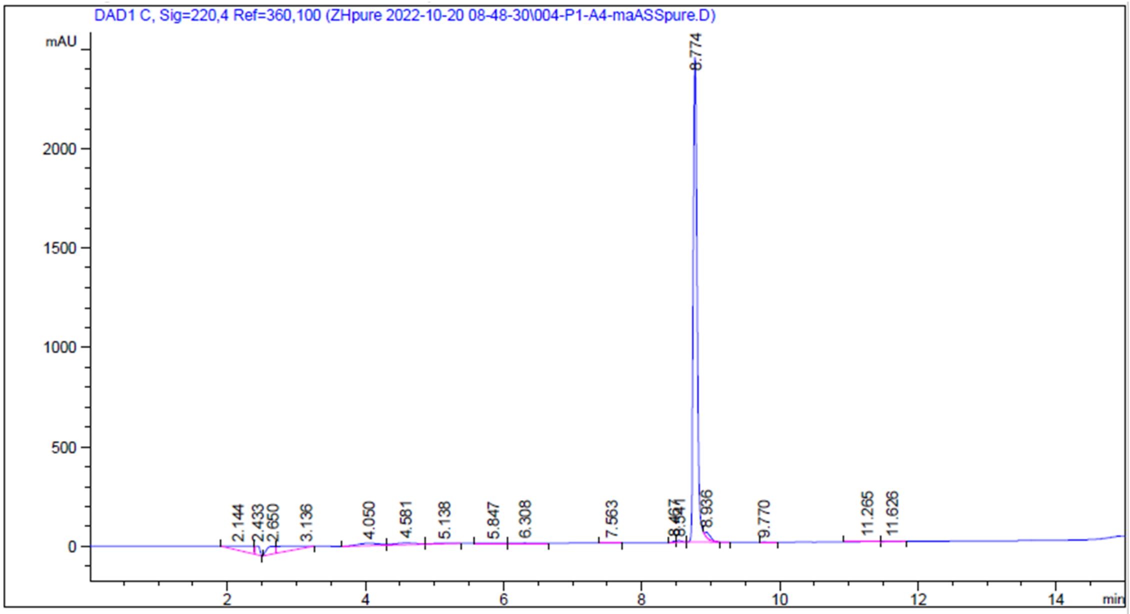

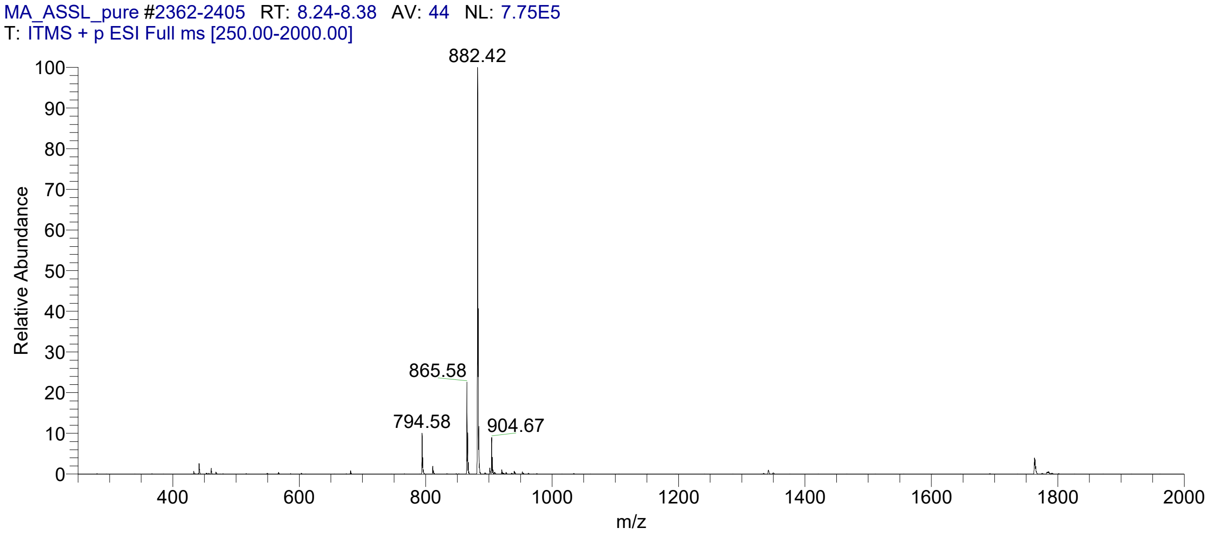

MPA-GHAIYPRH

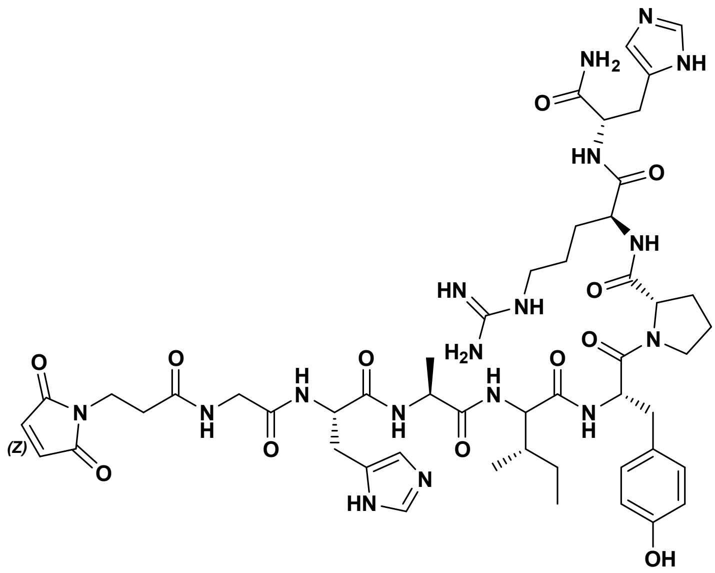

Exact Mass: 1099.53

Molecular Weight: 1100.21

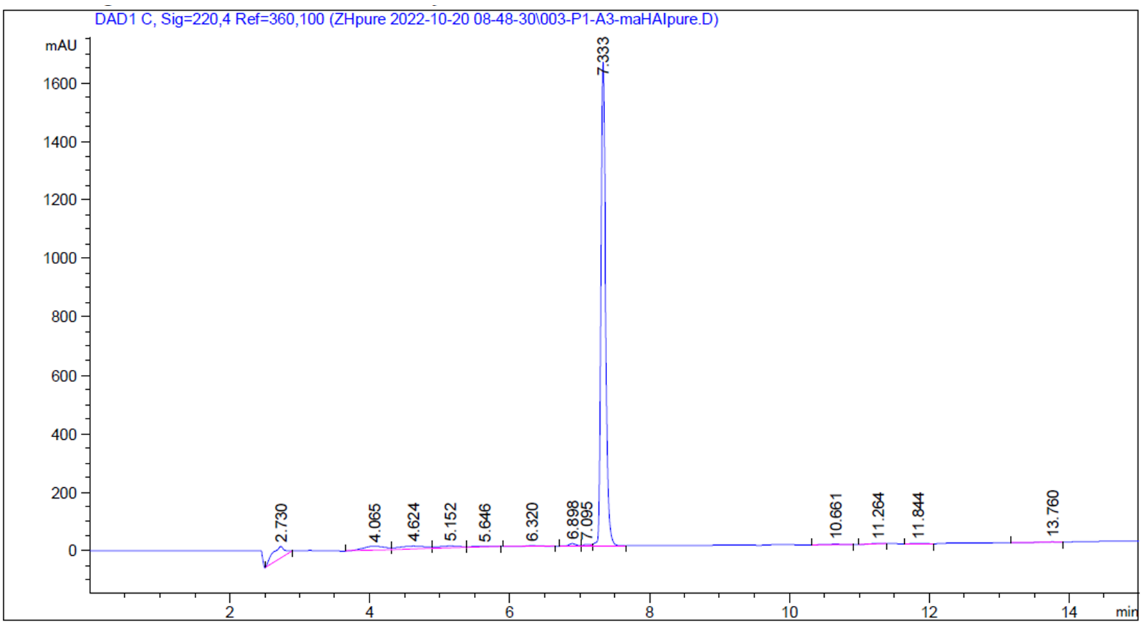

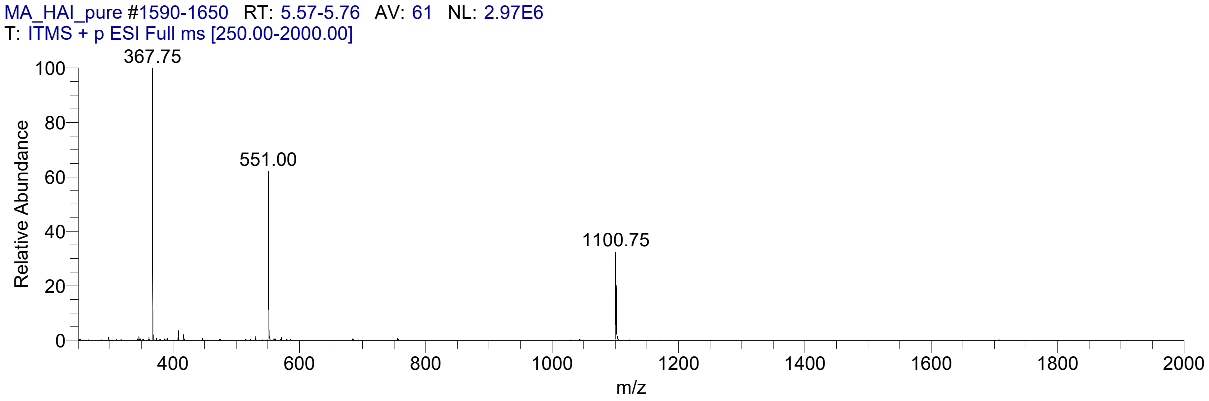

MPA-GGRGDSP

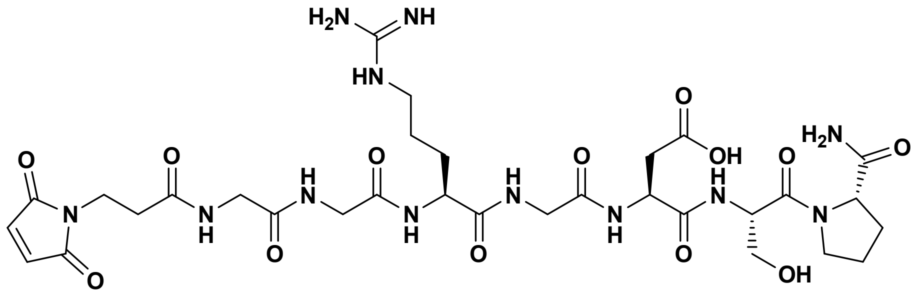

Exact Mass: 794.33

Molecular Weight: 794.78

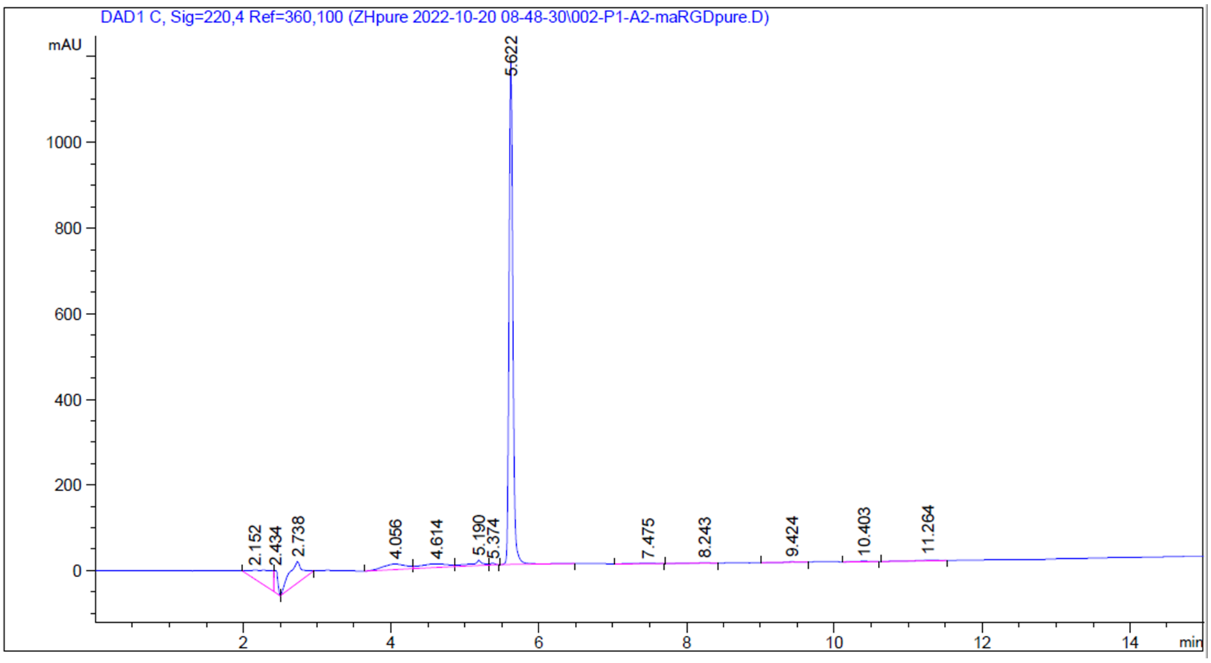

MA_RGD_pure #1441-1454 RT: 5.07-5.11 AV: 14 NL: 4.51E6
T: ITMS + p ESI Full ms [250.00-2000.00]

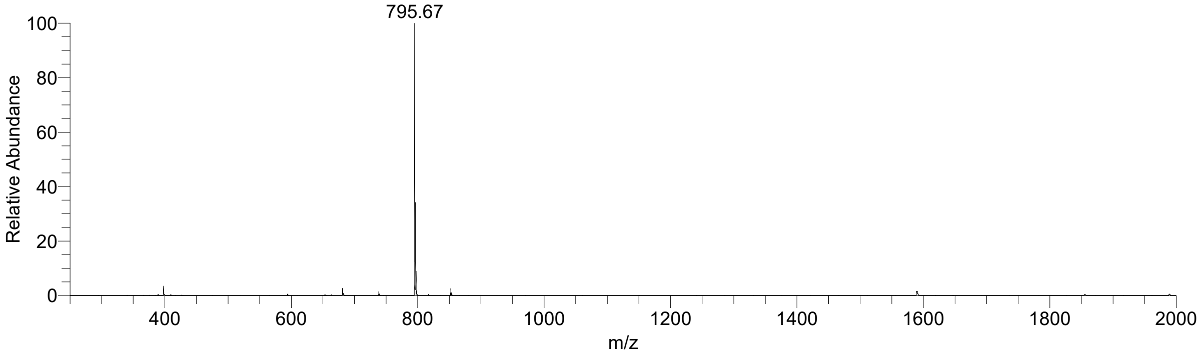

S2.3 LC-MS spectra of pure oligo-peptide conjugates

O1-GASSLNIA

MW: 4068.081; Expected: [M-3H]$^{3-}$: 1355.02; [M-4H]$^{4-}$: 1016.01; [M-5H]$^{5-}$: 812.61;

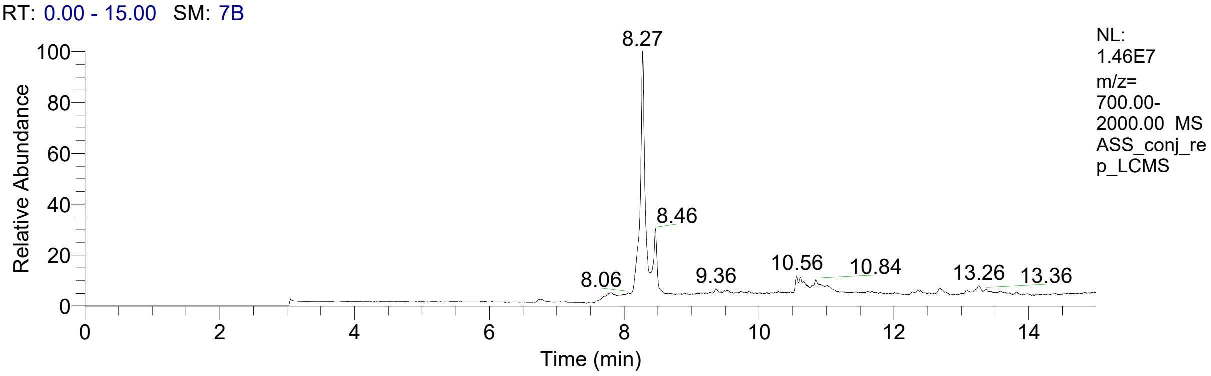

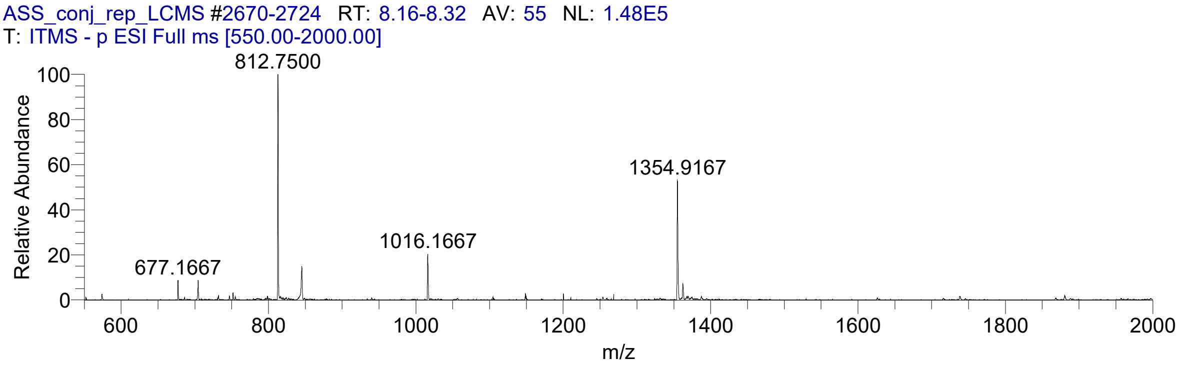

O2-GHAIYPRH

MW: 4237.103; [M-3H]$^{3-}$: 1411.50; [M-4H]$^{4-}$: 1058.50; [M-5H]$^{5-}$: 846.67;

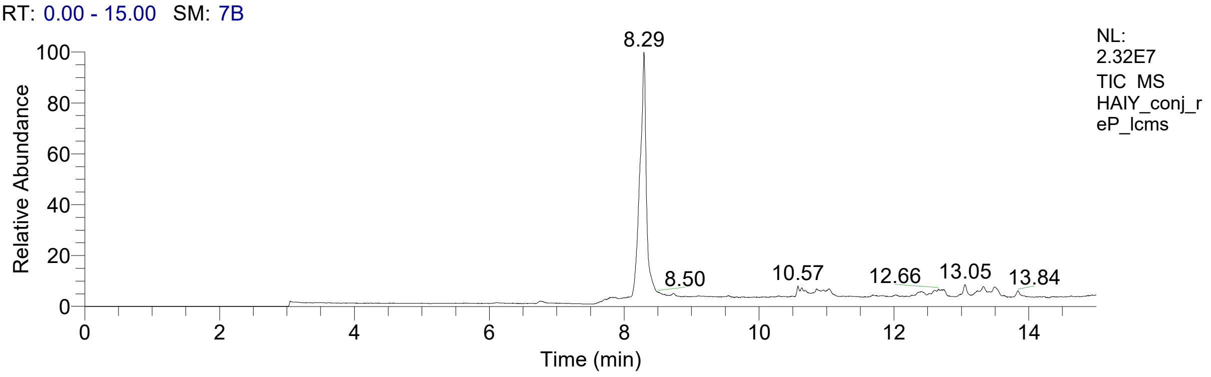

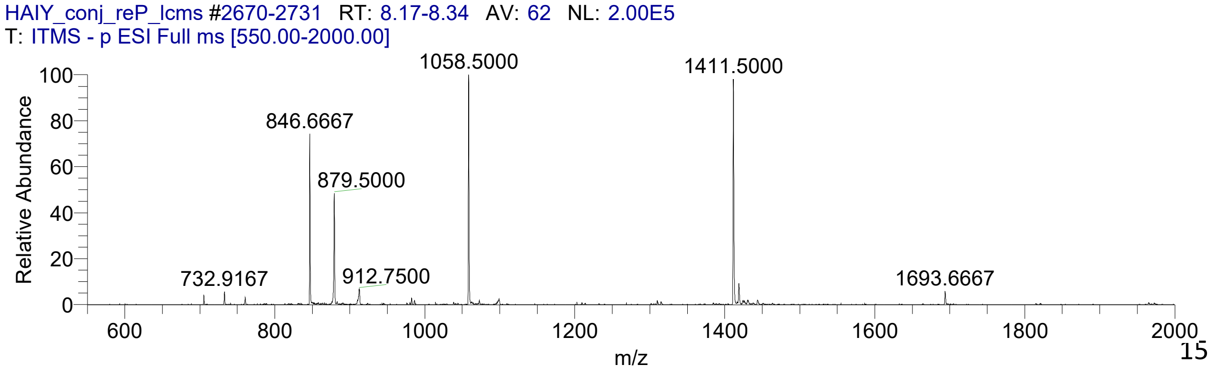

O3-GGRGDSP

MW: 3940.896, $[M-3H]^{3-}$: 1312.83; $[M-4H]^{4-}$: 984.41; $[M-5H]^{5-}$: 787.41;

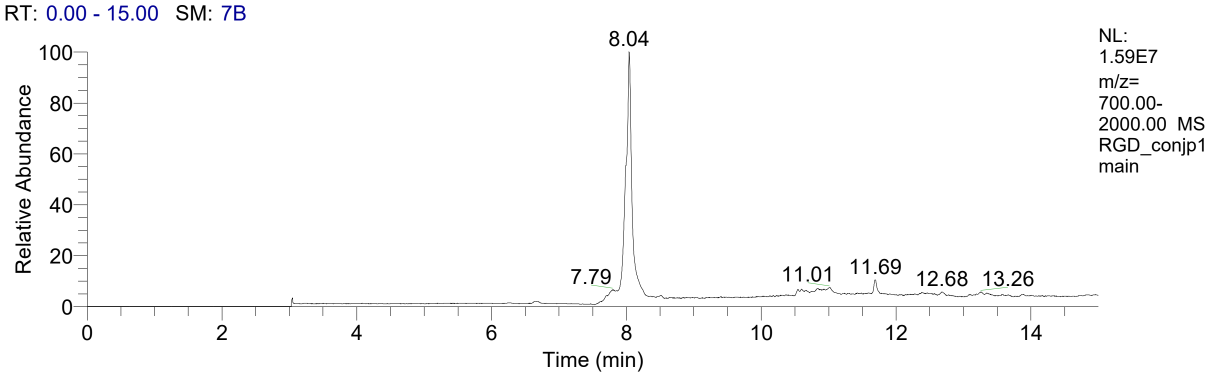

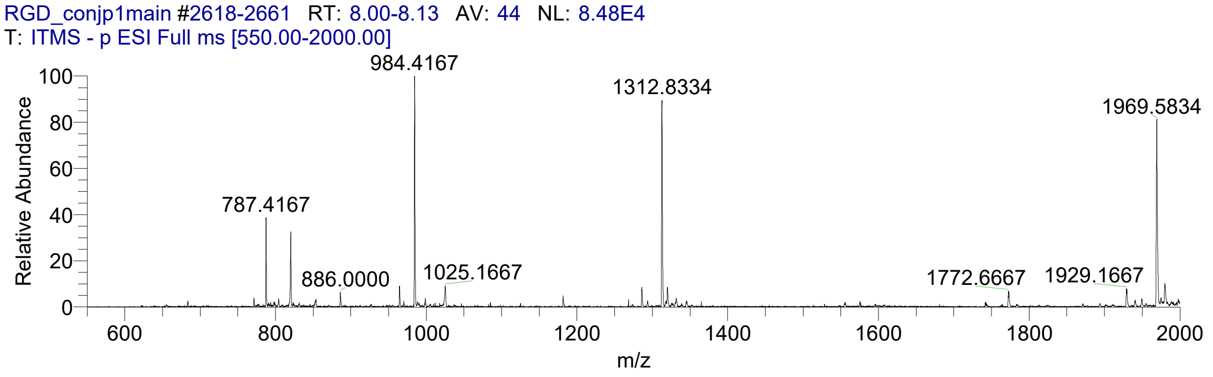

Supplement: Supplementary file 1 [file pharmaceutics-15-00248-s001.zip › pharmaceutics-2131994-supplementary.pdf]
